# Antibody‐Empowered Nanomedicine for Precise Biomedical Applications

**DOI:** 10.1002/advs.202521428

**Published:** 2026-01-12

**Authors:** Chen Chen, Xinglin Chen, Zhenhao Gao, Meng Sun, Yaozong Yu, Fang Lv, Qiujun Wang, Jinfeng Zhang

**Affiliations:** ^1^ Key Laboratory of Molecular Medicine and Biotherapy School of Life Science Beijing Institute of Technology Beijing P. R. China; ^2^ Department of Anesthesiology Hebei Medical University Third Hospital Shijiazhuang P. R. China

**Keywords:** antibody, active targeting, biomedical applications, immunonanoparticles, precision medicine

## Abstract

The advancement of nanotechnology has positioned nanomedicine as powerful tools for disease diagnosis and treatment. However, the clinical efficacy of conventional nanomedicine is often limited by its passive targeting strategies and limited range of therapeutic mechanisms, leading to suboptimal accumulation at diseased sites and compromised therapeutic outcomes. Fortunately, the incorporation of antibodies, with their unparalleled targeting specificity and immune‐activating capabilities, presents a pivotal strategy to overcome these hurdles of nanoparticles. This review systematically summarizes the current progress in antibody‐empowered nanomedicine. Different antibody functionalization strategies for nanoparticles, along with their respective advantages, limitations, and typical applications, are first discussed. This review then individually describes the classification of these nanomedicines based on antibody structures, such as IgG‐like antibodies, antibody fragments, and novel antibody‐nanomedicine fusion formats. Subsequently, a specific emphasis is placed on the highlight of their biomedical applications in diagnostics, bioimaging, and the treatment of various diseases. Finally, ongoing challenges and prospects in the clinical translation of antibody‐empowered nanomedicine are critically examined. This review is intended to catalyze interdisciplinary breakthroughs that propel antibody‐empowered nanomedicine as an important pillar of precision medicine.

## Graphical abstract

This review explores strategies for functionalizing nanoparticles with antibodies to construct antibody‐empowered nanomedicine. It discusses the classification of these nanomedicines based on antibody structure, with a specific focus on their biomedical applications in diagnostics, bioimaging, and therapeutics for various diseases. Finally, it critically examines the ongoing challenges and future prospects for the clinical translation of this technology.

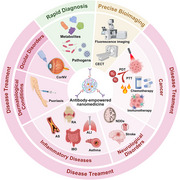

## 1 Introduction

With the rapid advancement of nanotechnology, nanomedicine has played a pivotal role in the diagnosis and treatment of diseases, providing innovative therapeutic strategies for various common medical conditions [1, 2, 3, 4, 5]. Nanomedicine refers to the functional materials with submicron‐scale dimensions, typically ranging from 1 to 100 nm [6, 7, 8]. The ultra‐small size grants them distinctive physiochemical and structural characteristics, leading to enhanced therapeutic effects [9, 10]. Concretely, by strategically modulating size and surface characteristics, these functional materials can avoid rapid clearance and achieve site‐specific drug delivery, while also enhancing drug solubility, stability, and bioavailability [11, 12, 13, 14]. Concurrently, nanomedicine can deliver diverse therapeutic agents (e.g., chemotherapeutics, biologics, and nucleic acids) and diagnostic imaging cargos, through tailoring composition [15, 16, 17]. Consequently, nanomedicine has been extensively utilized in a range of biomedical applications, including drug delivery, bioimaging, disease diagnostics, and treatment [18, 19, 20, 21, 22, 23].

Over the past few decades, significant breakthroughs in nanomedicine have culminated in the emergence of diverse classes, including polymeric, biomimetic, and inorganic nanoparticles [24, 25, 26]. Meanwhile, more than 500 nanomedicines are in clinical trials, promising to offer innovative solutions for the diagnosis and treatment of various diseases [27]. However, clinical trials of nanoparticle‐based therapies have demonstrated more limited efficacy than anticipated [28, 29, 30]. This limitation is primarily attributed to their mainly reliance on passive targeting mechanisms, which leverage intrinsic physicochemical properties of nanoparticle to achieve site‐specific accumulation, such as the enhanced permeability and retention (EPR) effect [31]. The randomness of drug diffusion and the inadequate diffusion to diseased sites lead to compromised therapeutic efficacy and the development of drug resistance [32, 33]. Moreover, the role of conventional nanomedicine as a passive drug carrier inherently defines its limited mechanistic scope for treatment [28]. Fortunately, the utilization of antibodies to engineer nanomedicine holds great potential to overcome these major drawbacks [14, 34].

Antibodies are well‐known specific targeted biomolecules, which play a crucial role in precision medicine. Antibodies have a basic Y‐shaped structure, comprising two antigen‐binding domains (Fab) and one crystallizable domain (Fc). The Fab region mediates the specific recognition between antibodies and antigens, which is central to the natural biological function of antibodies [35, 36, 37]. Given this function, antibodies can recognize antigens that are uniquely expressed or heavily overexpressed on diseased cells. Thus, the incorporation of antibodies enables nanoparticles to actively target diseased cells via antigen‐antibody recognition [38]. This active targeting strategy facilitates receptor‐mediated endocytosis and intracellular drug release, minimizing non‐specific interactions with healthy cells, reducing systemic toxicity in drug delivery, while enhancing diseased cells cytotoxicity [39, 40]. Meanwhile, the Fc region stands for immune effector functions, such as antibody‐dependent cellular cytotoxicity (ADCC), antibody‐dependent cellular phagocytosis (ADCP), and complement‐dependent cytotoxicity (CDC), to eliminate pathogens or diseased cells [41, 42, 43, 44]. By leveraging the Fc domain, antibodies can endow nanoparticles with immunotherapeutic capabilities, thereby significantly boosting therapeutic efficacy and broadening their application scope [40].

**FIGURE 1:**
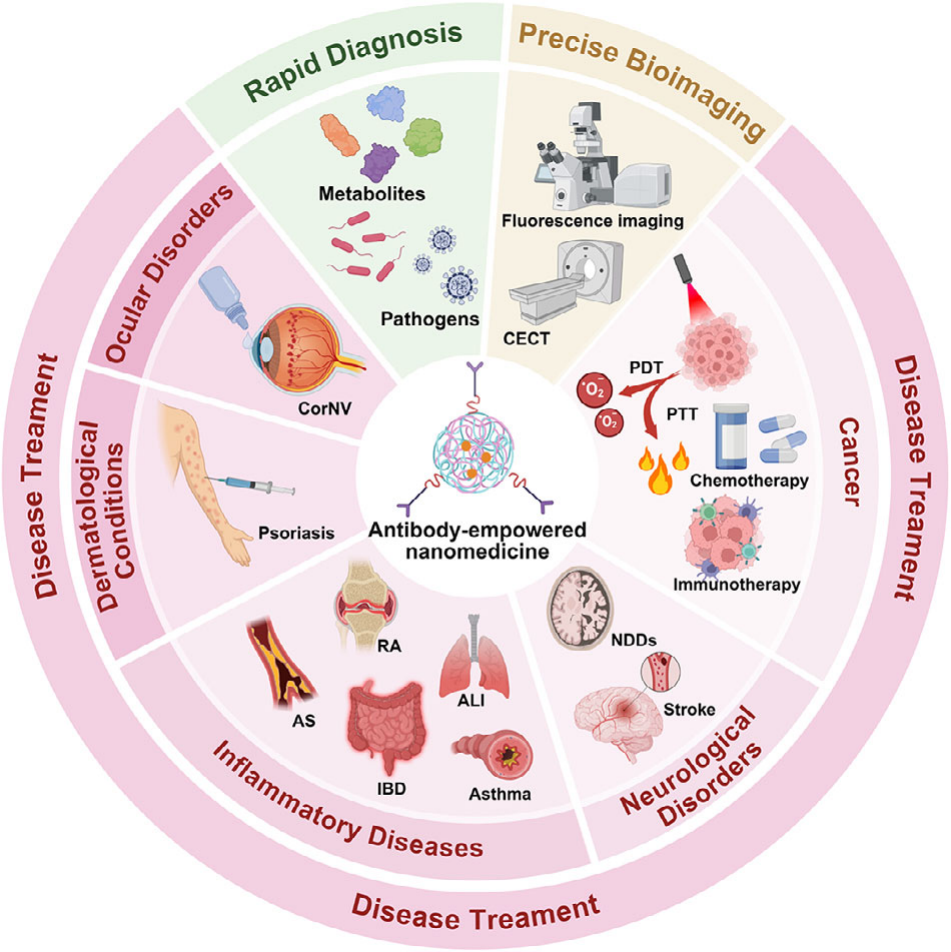
Schematic illustration of the antibody‐empowered nanomedicine for various precise biomedical applications. Figure created with BioRender.com.

**FIGURE 2:**
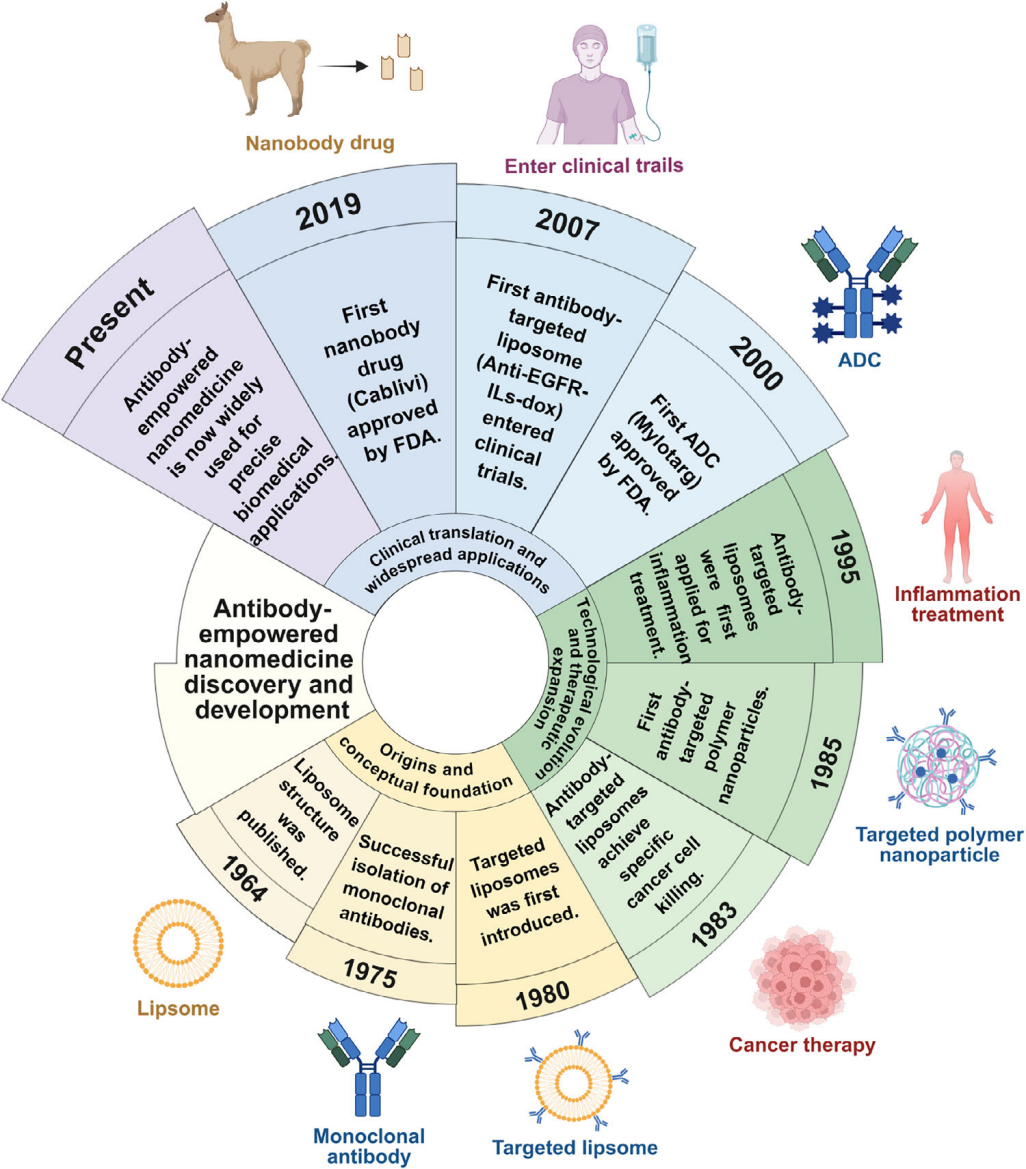
Timeline of key developmental milestones of antibody‐empowered nanomedicine [40, 45, 46, 47, 48, 49, 50, 51, 52, 53, 54]. Figure created with BioRender.com.

In this review, we systematically summarize the recent progress in the development of antibody‐empowered nanomedicine from three aspects, including: 1) antibody functionalization strategies for nanomedicine along with their respective advantages, limitations, and typical applications, 2) classification of antibody‐empowered nanomedicine based on antibody structures, and 3) recent advances in the applications of antibody‐empowered nanomedicine for diagnostics, bioimaging, and therapy (Figure 1). Meanwhile, key developmental milestones of antibody‐empowered nanomedicine are summarized in Figure 2. Additionally, we discuss the challenges and prospects in this field, by critically examining the persisting obstacles hindering the clinical translation and exploring promising future innovation pathways. Through interdisciplinary collaboration, antibody‐empowered nanomedicine is well‐positioned to achieve clinical breakthroughs in the coming decades, advancing precision medicine through highly targeted and intelligent therapeutics.

## 2 Antibody Functionalization Strategies for Nanomedicine

**FIGURE 3:**
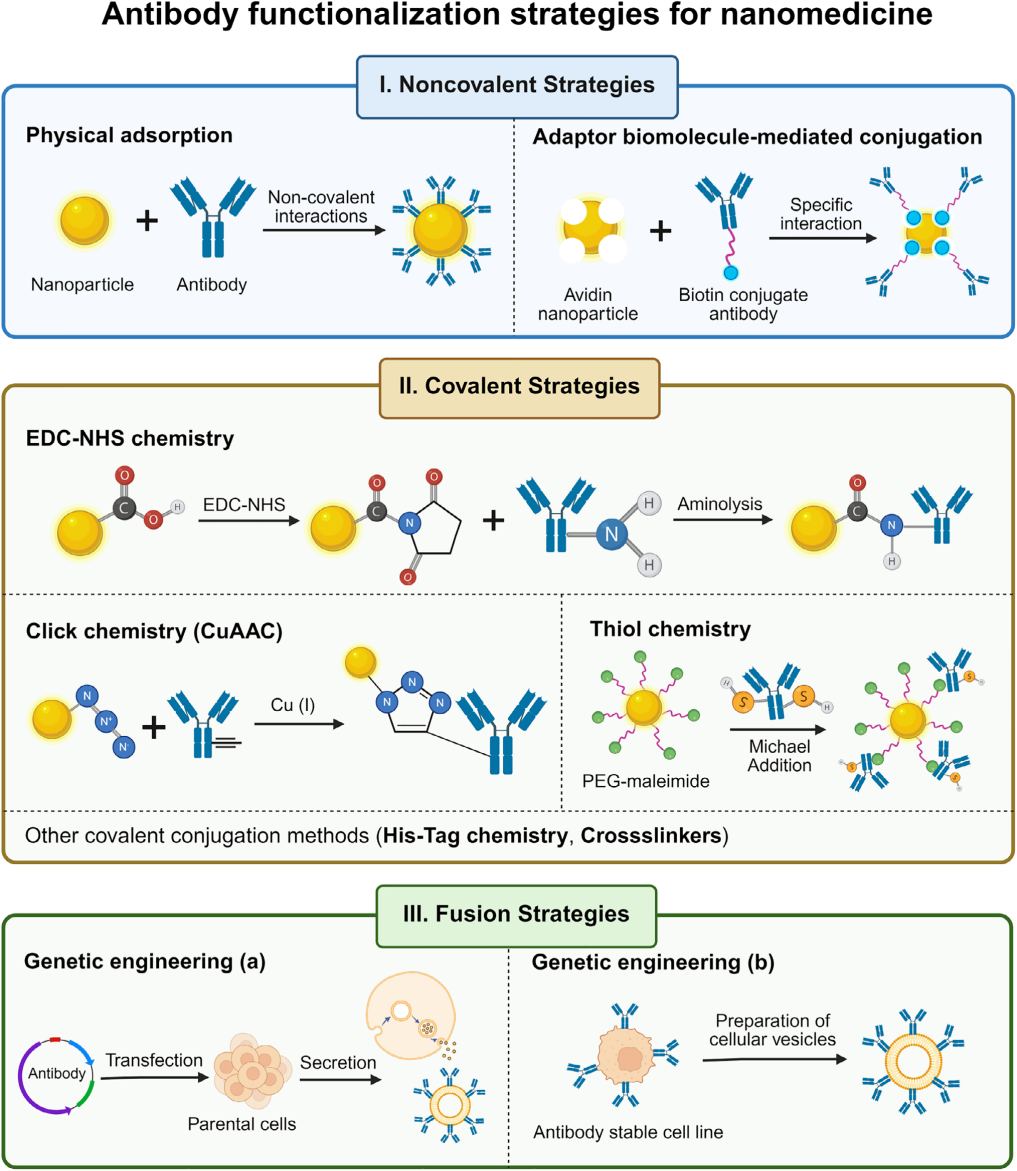
Schematic illustration of three main antibody functionalization strategies for nanomedicine, including noncovalent strategies, covalent strategies, and fusion strategies. Figure created with BioRender.com.

Antibody functionalization strategies for nanomedicine refer to the surface modification of nanoparticles via antibody conjugation to improve targeting and therapeutic efficiency [40, 55]. This process requires stable binding to minimize off‐target toxicity, proper antibody orientation to ensure targeting capability, and prevention of antibody aggregation during conjugation [56, 57]. The current major functionalization strategies can be categorized as noncovalent, covalent, and novel fusion strategies (Figure 3), and their respective advantages, limitations, and typical applications are summarized in Table 1.

**TABLE 1:** Summary of the advantages, limitations, and typical applications of different antibody functionalization strategies for nanomedicine.

Strategy		Advantages	Limitations	Typical applications	Refs.
**Noncovalent strategies**	Physical adsorption	Simple operation; Minimal disruption to antibody structure and function; Suitable for various antibodies and nanomaterials	Unstable binding; Difficulty in controlling antibody orientation; Susceptible to protein corona effect	Proof‑of‑concept studies; In vitro diagnostics; Short‐term use	[57, 58, 62, 63]
	Biomolecule‐mediated conjugation	Specific and oriented antibody immobilization	Potential immunogenicity; Formation of heterogeneous conjugates; Possible dissociation between proteins and antibodies	Oriented immobilization without covalent modification	[66]
**Covalent strategies**	EDC‐NHS chemistry	Most prevalent method; Forms stable amide bonds; Relative simplicity and broad applicability	Random orientation; Potentially hindering antigen binding; Promotes antibody self‐aggregation	General use; Stability prioritized over orientation	[70, 71, 72, 73]
	Click chemistry	Enables site‐specific attachment; High specificity and yield, often without complex purification, Copper‐free variants avoid copper cytotoxicity	Requires pre‐functionalization of both components; Relative complexity of synthesis steps; CuAAC limited by copper catalyst cytotoxicity	Site‐specific conjugation for maximal antigen binding	[77, 82, 83]
	Thiol chemistry	Enables site‐specific conjugation; Minimizes obstruction of the Fab domains	Promotes antibody self‐aggregation; Potential to adversely affect antibody folding and stability	Engineered antibody fragments (e.g., scFv, nanobodies)	[84, 85, 88]
	His‐Tag chemistry	Offers directional binding and reversibility	Metal ion leakage affects biocompatibility; Reduced stability in serum	Lab‐scale purification and immobilization	[40]
	Crosslinkers	Flexible tools for bridging groups	Homobifunctional linkers cause random conjugation; Process complexity; Raises immunogenicity concerns	Custom linkage; Limited clinical suitability	[89]
**Fusion strategies**	Genetic engineering	Inherently targeted nanoconstructs; Avoids incorrect orientation and structural distortion of antibodies; Preserves biological functionality of native membrane proteins; Improves sustainability and stability	Introduction of exogenous proteins may disrupt cellular homeostasis; Potential to provoke immune responses; Donor cells may face metabolic burden or unintended signaling’ Lack of standardized protocols	Native‐like presentation; Experimental/therapeutic nanovesicles	[90, 91, 92]

### 2.1 Noncovalent Strategies

Noncovalent strategies primarily comprise physical adsorption and biomolecule‐mediated conjugation. Physical adsorption enables antibodies bind to the surface of nanoparticles through noncovalent interactions, such as hydrogen bonds, hydrophobic interactions, electrostatic forces, and van der Waals interactions. Compared with covalent conjugation, this approach requires no chemical modification, is easy to operate, minimizes disruption to the structure and function of antibodies, and is suitable for various types of antibodies and nanomaterials [57, 58]. For example, Huang et al. demonstrated a simple and efficient antibody conjugation strategy based on hydrogen bonding, which avoided chemical modification while maintaining the structural integrity [59]. Similarly, Li et al. developed galloylated liposomes (GA‐lipo) that adsorb antibodies through hydrophobic interactions and hydrogen bonds, a mild process that preserves antibody functionality without the need for complex coupling chemistry [60]. In addition, the assembly of antibody‐targeted lipid nanoparticles (Ab‐LNPs) often relies on straightforward electrostatic adsorption between antibody residues and the nanoparticle surface, offering a versatile and generally applicable method that retains antibody activity [61].

Despite these advantages, physical adsorption is hampered by several notable limitations, including unstable binding that is vulnerable to environmental parameters like ionic strength and pH, difficulty in controlling antibody orientation that may affect antigen‐binding activity, and susceptibility to the protein corona effect with competitive adsorption of serum proteins further weakening binding efficacy [62, 63]. For instance, Dai et al. reported that the protein corona derived from human serum (HS) suppressed the specific targeting association of nanoparticles from approximately 70% to about 7% [64]. Archontakis et al. found that the random orientation of antibodies leads to significant single‐particle heterogeneity within the same batch of nanoparticles, and the functional‐to‐total antibody ratio decreases as the concentration of conjugated antibodies increases [65]. These limitations have driven the development of alternative conjugation strategies designed to precisely control antibody orientation and enhance stability against biological interference, such as click chemistry, thiol chemistry, and genetic engineering. In conclusion, noncovalent strategies are particularly suitable for proof‑of‑concept studies, in vitro diagnostics, or short‑term applications where simplicity and minimal antibody modification are prioritized over long‑term stability.

In addition to physical adsorption, adaptor biomolecule‐mediated conjugation represents another prominent noncovalent strategy. It conjugates antibodies with nanoparticles by utilizing specific high‐affinity interactions between biomolecules, such as avidin‐biotin and protein A/G‐Fc binding. This strategy thus enables specific and oriented antibody immobilization by exploiting the asymmetric structure of antibodies, typically through selective binding to the conserved Fc region [66]. For example, Schiavon et al. conjugated cetuximab monoclonal antibody with DM1 derivative‐based nanoparticles by leveraging the avidin‐biotin interaction, where the biotin moiety was site‐specifically attached to the Fc region of antibody, ensuring the antigen‐binding Fab domains remain accessible [67]. And, Liang et al. functionalized silica‐coated upconversion nanoparticles (UCNPs) by employing a Linker‐Protein G fusion. The high affinity of Protein G for the antibody Fc region guaranteed a uniform orientation with the Fab arms directed outward [68]. Ko et al. further demonstrated a modular approach by functionalizing gold nanoparticles with a genetically engineered Gold‐Binding‐Polypeptide‐Protein A (GBP‐ProA) fusion. Here, the GBP domain anchored the construct to the nanoparticle surface, while the Protein A domain provided the oriented, Fc‐specific capture of antibodies [69]. While these adaptor‐based systems effectively address the orientation challenge inherent to simple adsorption, they are not without limitations. A primary concern is the potential immunogenicity of the adaptor molecules themselves (e.g., bacterial‐derived Protein A/G or avidin), which could trigger unintended immune responses in vivo. Other drawbacks include the formation of heterogeneous conjugates due to multivalent binding sites and the possibility of dissociation between the adaptor and the antibody under certain conditions [66]. Consequently, this approach is typically chosen for oriented immobilization when covalent conjugation is technically challenging, risks compromising antibody integrity, or is otherwise impractical.

### 2.2 Covalent Strategies

Covalent conjugation provides a stable strategy for immobilizing antibodies onto nanoparticles through the formation of irreversible chemical bonds between functional groups on the nanoparticle and the antibody [70]. This approach can be broadly categorized based on the reactive groups involved, including EDC‐NHS chemistry, thiol‐maleimide chemistry, click chemistry, His‐tag chemistry, and crosslinkers.

EDC‐NHS chemistry is the most prevalent method for conjugating antibodies to carboxylic acid‐functionalized nanoparticles (e.g., liposomes, polymeric NPs) via stable amide bonds. The popularity of EDC‐NHS chemistry stems from its simplicity and broad applicability [71, 72, 73]. These characteristics are reflected in its widespread use across diverse studies. For example, Saha et al. leveraged the EDC‐NHS chemistry to functionalize nanoparticles with anti‐CD47 and anti‐PD‐L1 antibodies, demonstrating its utility in immune‐modulating nanotherapeutics [74]. Similarly, the versatility is shown by Ceylan et al., who coupled the VEGF165 antibody to LpAB‐FeNPs for specific imaging, and by Cakir et al., who conjugated the anti‐VEGF antibody Bevacizumab to carboxylated niosomes for therapeutic applications [75, 76]. In these and many other cases, EDC‐NHS chemistry provides a reliable and accessible route to achieve covalent linkage without requiring highly specialized expert. However, a significant drawback is the random orientation of the conjugated antibody. Since amine groups are distributed across the antibody surface (including on Fab domains), the conjugation reaction occurs indiscriminately. This can result in a substantial proportion of antibodies being immobilized with their Fab regions facing downward or sideways against the nanoparticle surface, which severely compromises their antigen‐binding capability. Consequently, a large fraction of the conjugated antibodies is functionally inactive, necessitating higher antibody loading to achieve the desired targeting effect. This not only increases material costs but may also elevate the risk of off‐target toxicity and immunogenicity. Additionally, the non‐specific nature of the reaction can promote antibody self‐aggregation [70]. These limitations inherent to random conjugation highlight the critical need for site‐specific strategies to fully harness the therapeutic potential of antibody‐nanoparticle conjugates. While these limitations underscore the need for advanced site‐specific strategies, EDC‑NHS chemistry remains widely adopted for its ease of use and robustness, making it a practical choice for many therapeutic nanoparticle formulations where high stability is required, even if some loss of activity due to random orientation is acceptable.

To overcome the orientation challenge inherent in random conjugation (e.g., EDC‐NHS chemistry), site‐specific strategies such as click chemistry have been developed. Among them, copper‐catalyzed azide‐alkyne cycloaddition (CuAAC) offers a highly efficient and bioorthogonal route. This reaction requires pre‐modifying nanoparticles and antibodies with complementary functional groups (e.g., azide and alkyne), which react under copper catalysis to form a stable triazole linkage, enabling highly specific coupling with minimal side reactions [77]. The efficiency and specificity of CuAAC are illustrated in its application for targeted nanoconstructs. For example, Asad et al. conjugated azide‐modified antibodies to alkyne‐functionalized SPIONs via CuAAC, achieving precise coupling for targeted imaging and therapy of inflammatory bowel disease [78]. Similarly, Kuo et al. utilized the same chemistry to covalently link alkynylated antibodies to azide‐modified Cu_2_O@Ag nanoplates, demonstrating reliable and controlled orientation in a plasmonic nanoplatform [79]. However, the cytotoxicity of copper catalysts limits the in vivo applicability of CuAAC. To address this, copper‐free alternatives such as strain‐promoted azide‐alkyne cycloaddition (SPAAC) and strain‐promoted azidenitrone cycloaddition (SPANC) have been introduced, retaining bioorthogonality while improving biocompatibility. For instance, Escudé et al. immobilized azide‐modified nanobodies onto DBCO‐functionalized LNPs via the copper‐free SPAAC reaction, showcasing a biocompatible route for oriented conjugation [80]. Colombo et al. further employed SPANC to site specifically conjugate an antiHER2 scFv to multifunctional nanoparticles, underscoring the utility of copper‐free click chemistry in maintaining antibody functionality [81]. Despite these advantages, the broader adoption of click chemistry is constrained by the need for prefunctionalization and relatively complex synthesis steps [82, 83]. Nonetheless, click chemistry is preferentially employed when site‐specific, oriented conjugation is essential for preserving high antigen binding activity, especially in advanced nanomedicine designs where synthetic control outweighs operational complexity.

Another important strategy for achieving directional conjugation is thiol chemistry. This approach utilizes thiol groups (‐SH) on antibodies, either natively present or introduced via engineering, to react with maleimide‐functionalized nanoparticles. This site‐specificity minimizes obstruction of the Fab domains and preserves antigen‐binding activity [84, 85]. The practical application of this strategy is demonstrated in studies utilizing engineered antibody fragments. For instance, Mohamed et al. introduced active thiol functionality into CRLF2‐targeting single‐chain variable fragment (CRLF2 scFv‐Fc), and conjugated it to a maleimide‐functionalized liposomes, generating CRLF2‐targeted liposomes (CRLF2‐DM1 LIP) [86]. Jung et al. conjugated CD11c nanobodies to nanocarriers via a C‐terminal cysteine‐mediated thiol‐maleimide reaction [87]. Notably, key considerations for thiol chemistry include maintaining a reducing environment to prevent disulfide‐mediated aggregation and ensuring that the introduction of such functional groups does not impair antibody folding or stability [88]. Thiol‑based conjugation is favored for recombinant antibody fragments (e.g., scFvs, nanobodies) that can be engineered with terminal cysteines, offering a good balance between site‑specificity, stability, and scalability for clinical‑grade production.

Other covalent conjugation methods serve specific niches. For instance, His‐Tag chemistry exploits the high‐affinity coordination between polyhistidine tags, which often genetically fused to recombinant antibody fragments like scFvs, and transition metal ions (e.g., Ni^2^^+^, Co^2^^+^) immobilized on nanoparticles. While offering directional binding and reversibility (e.g., by imidazole elution), concerns include potential metal ion leakage affecting biocompatibility and reduced stability in complex biological fluids like serum [40]. Histag chemistry is mainly useful for laboratory‐scale purification and immobilization, but its in vivo application is limited due to stability and safety considerations. Bifunctional crosslinkers represent another classic conjugation strategy. They can be categorized into homobifunctional types (e.g., glutaraldehyde, DSS) and heterobifunctional types (e.g., SPDP, GMBS), which bridge diverse functional groups (e.g., amines, thiols, carboxyls) on antibodies and nanoparticles. Heterobifunctional crosslinkers in particular allow controlled, directional coupling. Nevertheless, several limitations persist, homobifunctional variants often result in random conjugation and activity loss, some crosslinkers (e.g., glutaraldehyde) raise immunogenicity concerns, and the multistep conjugation process can be complex, hampering reproducibility in large‐scale manufacturing [89]. Crosslinker‑based methods are typically employed when custom linkage between specific functional groups is needed, but they are less favored for clinical translation due to potential immunogenicity and process variability.

### 2.3 Fusion Strategies

Beyond conventional conjugation methods, a genetic engineering strategy has emerged as a powerful alternative for creating inherently targeted nanoconstructs. This approach involves genetically modifying parent cells to express antibody directly on their membranes, from which targeted biosynthetic nanovesicles (e.g., membrane vesicles or extracellular vesicles) are then derived [90, 91]. This strategy inherently ensures correct antibody orientation and preserves biofunctionality, overcoming key limitations of chemical conjugation [92]. For instance, Xue et al. engineered macrophages to display PD‐1‐specific scFvs on their surface, yielding derived nanovesicles that effectively suppressed tumor growth in mice, showcasing how the native display preserves the functional conformation of scFvs for immune checkpoint blockade [93]. Similarly, Gao et al. generated extracellular vesicles (EVs) decorated with PSMA‐targeting scFvs via cellular engineering, resulting in a platform that enabled precise prostate cancer therapy, as the correctly oriented scFvs maintained high targeting specificity [94]. Furthermore, the versatility of this approach is highlighted by Liu et al., who constructed a multifunctional delivery system by engineering cell membrane vesicles to display full‐length antibodies, proving that complex proteins can be functionally integrated using this methodology [95]. However, genetic engineering faces significant challenges. The introduction of exogenous proteins can disrupt cellular homeostasis and provoke immune responses, donor cells may experience metabolic burden or unintended signaling, and the lack of standardized protocols hinders reproducibility and clinical translation [90, 91].

To address these challenges, several countermeasures are being explored. The immunogenicity and cellular disruption caused by exogenous protein expression can be mitigated by using human‐derived or engineered stealth sequences and by employing inducible expression systems to minimize metabolic burden. Regarding standardization, key aspects requiring harmonization include, the selection and engineering of parental cell lines (e.g., HEK‐293 for low immunogenicity), standardized methods for vector design and transfection to ensure consistent antibody display, and the establishment of unified quality control metrics for the resulting nanovesicles, such as antibody surface density, vesicle size distribution, and targeting functionality [90, 91]. Given these considerations, fusion strategies represent a next‐generation approach, ideally suited for creating complex, biologically active nanovesicles where natural antibody presentation and cellular homing properties are critical. They are particularly promising for immunotherapy and targeted drug delivery, yet their clinical translation will depend on overcoming the aforementioned challenges through continued technological refinement and standardization efforts.

## 3 Classification of Antibody‐Empowered Nanomedicine Based on Antibody Structures

**FIGURE 4:**
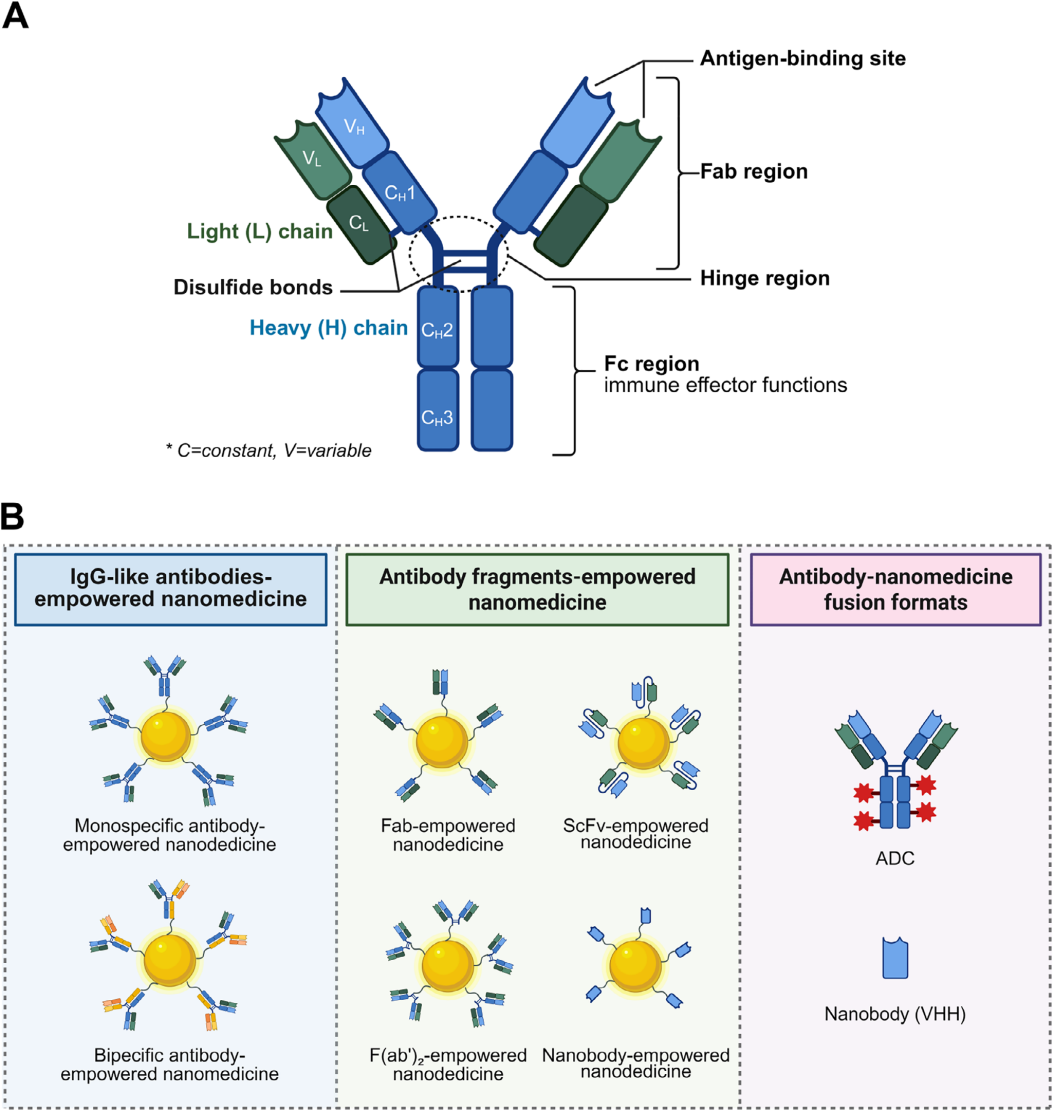
Schematic illustration of A) the structure and function of IgG, and B) three main categories of antibody‐empowered nanomedicines. Figure created with BioRender.com.

Owing to the inherent heterogeneity across diseases, including manifested in variations of genetic profiles, dysregulated cellular pathways, and distinct microenvironmental factors, no single optimal antibody format exists for all therapeutic applications [96]. Fortunately, advances in antibody engineering have led to a diverse repertoire of antibody formats, each offering unique structural and functional properties that can be leveraged for the development of targeted nanomedicines. Based on antibody structural features, antibody‐empowered nanomedicines can be broadly classified into three main categories, including IgG‐like antibody‐empowered nanomedicine, antibody fragments‐empowered nanomedicine, and novel antibody‐nanomedicine fusion formats. Figure 4A illustrates the basic structural features and biological functions of conventional immunoglobulin G (IgG) antibodies, while Figure 4B presents the three main categories of antibody‐empowered nanomedicines described above.

### 3.1 IgG‐Like Antibodies‐Empowered Nanomedicine

IgG‐like antibodies retain the canonical Y‐shaped structure of immunoglobulin G (IgG), comprising Fab domains for antigen binding and an Fc domain responsible for immune effector functions. These antibodies are increasingly employed to functionalize nanomedicines, enhancing targeting precision and therapeutic efficacy. Based on antigen‐binding valency, IgG‐like antibodies can be classified into three main formats, including monospecific, bispecific, and trispecific antibodies [44].

The large‐scale production of monospecific antibodies was first achieved through hybridoma technology, pioneered by Milstein and Köhler in 1975 [46]. Monospecific antibodies can recognize a single antigenic epitope, their high specificity minimizes off‐target effects and facilitates targeted delivery of nanotherapeutic agents to diseased cells, making them a cornerstone of antibody‐based therapies [97, 98, 99, 100]. Therefore, monospecific antibodies are particularly suitable when the disease mechanism is driven primarily by a single, well‐defined molecular target. For example, Penon et al. developed anti‐erbB2 antibody‐conjugated gold nanoparticles for targeted photodynamic therapy against erbB2‐overexpressing SK‐BR‐3 breast cancer cells [101]. Shi et al. developed a tumor vasculature‐targeted system by engineering nanoparticles with the TRC105 antibody specific for the CD105 receptor [102]. Beyond this precise targeting conferred by the Fab region, the conserved Fc domain of antibodies presents a distinct and exploitable advantage for nanomaterial engineering. For instance, Alt et al. developed a strategy for fabricating antibody‐decorated metal‐organic framework (MOF) nanocrystals through the electrostatic anchoring of the negatively charged antibody Fc region to the MOF matrix, thereby ensuring the Fab regions remained exposed for targeting [103]. Despite their widespread use, a key limitation lies in their monospecificity, since disease progression often involves multiple molecular pathways, targeting only one antigen fundamentally restricts their therapeutic potential [104, 105].

To overcome the limitation of targeting a single antigen, researchers have turned to bispecific antibodies (bsAbs) for functionalizing nanomedicines. By engaging two different epitopes, bsAbs confer multifunctional targeting capabilities to nanocarriers, enabling sophisticated therapeutic mechanisms [106, 107]. Thus, bsAbs are the format of choice when dual targeting is required to enhance specificity, overcome resistance, or modulate the microenvironment. For instance, Xu et al. developed a nanosystem functionalized with a bispecific antibody (SS‐Fc) that simultaneously targets carcinoembryonic antigen (CEA) on cancer cells and CD16 on NK cells, thus enhancing tumor targeting and activating cytotoxic immunity [108]. Similarly, Liu et al. developed a bispecific antibody‐functionalized nanodelivery system that coordinately modulates both stromal and tumor cells in the tumor microenvironment for synergistic therapy [109]. In addition to enhanced targeted and therapeutic efficacy, bsAbs also offer versatile utility in innovative fabrication strategies. For instance, Dietmair et al. developed a platform where bsAbs serve as non‐covalent bridges, redirecting unmodified mRNA‐LNPs to specific cells (e.g., EGFR+/PSMA+) without altering the properties of nanoparticle, offering a flexible targeting solution [110].

Trispecific antibodies (TsAbs) can engage three distinct antigens, allowing even more sophisticated therapeutic interventions, such as simultaneous immune cell activation and dual pathway blockade. This multi‐target approach is conceptually promising for complex diseases requiring integrated intervention, though the structural complexity means development remains in early stages [111]. By conjugating these advanced antibody formats to nanocarriers, it is possible to achieve highly selective drug delivery, improved pharmacokinetics, and enhanced therapeutic outcomes across a range of diseases.

### 3.2 Antibody Fragments‐Empowered Nanomedicine

Antibody fragments, which refer to smaller functional units derived from immunoglobulins via proteolytic digestion or recombinant DNA technology, have emerged as another powerful tools for enhancing nanomedicine delivery. A key characteristic of these fragments is the presence of at least one Fab domain while lacking the Fc region. This structural feature minimizes off‐target interactions and improves pharmacokinetic properties, thereby enabling targeted drug delivery with improved specificity and efficiency. They are particularly suitable when the therapeutic objective requires deep tissue penetration, reduced immunogenicity, or minimized Fc‐mediated off‐target effects, while retaining antigen‐binding specificity [42].

The most widely used antibody fragments are Fab (∼47–48 kDa), consisting of a full light chain and part of the heavy chain connected by a disulfide bond. The monovalent binding and absence of the Fc region in Fab fragments minimize nonspecific interactions, making this format the choice for applications demanding high targeting precision without effector function or avidity effects. For example, Hou et al. constructed a theranostic nanoprobe by conjugating the Fab fragment onto nanoparticles to enhance targeting of fibroblastic foci [112]. Li et al. controllably immobilized anti‐HER2 Fab‐6His onto silica nanoparticles (Si NPs) via a site‐directed approach, enabling enhanced drug delivery to HER2‐positive breast cancer cells [113]. Another important antibody fragments format is the bivalent F(ab’)_2_ (∼110 kDa), which comprises two linked Fab units. Divalency of F(ab’)_2_ allows simultaneous engagement with two epitopes, resulting in higher avidity and the ability to cross‐link antigens. Therefore, F(ab’)_2_ fragments are suitable when stronger antigen retention or receptor clustering is desired, bridging the specificity of fragments with the avidity of full antibodies. For instance, Kim et al. reprogrammed T cell metabolism by engineering nanoparticle surfaces with anti‐CD3e F(ab’)_2_ fragments that specifically engage the CD3 receptor on T cells [114].

Moving to even smaller recombinant formats, single‐chain variable fragments (scFvs, ∼25 kDa) fuse the variable regions of heavy and light chains via a peptide linker. This design combines minimal size with high expression yield and ease of genetic modification. The scFvs format is highly amenable to nanoconjugation and genetic fusion strategies, making them ideal for constructing multifunctional nanoplatforms where engineering flexibility is paramount. For instance, Chen et al. used scFvs to functionalize antiferromagnetic nanoparticles, the constructed nanoprobe can selectively bind alpha‐synuclein oligomers [115]. Kang et al. further capitalized on the genetic flexibility of scFvs by fusing them to bacterial lipoproteins, enabling their spontaneous “plug‐and‐display” incorporation into lipid nanoparticle bilayers [116].

Representing an even more compact format, nanobodies (VHH, 12–14 kDa) are minimal binding domains derived from camelid heavy‐chain antibodies. Their combination of minimal size, remarkable stability, and high affinity positions them as superior targeting ligands for nanomedicine, particularly in applications requiring extreme tissue penetration or stability in harsh conditions. For instance, Qu et al. designed semiconducting polymer nanoparticles engineered with a midkine nanobody for precise photodynamic therapy, where the small size of nanobody promotes enhanced tumor penetration [117]. In another study, the high affinity and stability of anti‐PSMA nanobodies were exploited to decorate lipid nanoparticles (LNPs), enabling targeted mRNA delivery to prostate cancer cells and offering a potential therapeutic strategy for castration‐resistant prostate cancer (CRPC) [80].

However, it is important to acknowledge several inherent limitations of nanobodies that currently constrain their broader application. A primary challenge lies in their production, unlike conventional monoclonal antibodies derived from mice, nanobodies are typically sourced from immunized camelids or sharks, a process that is more complex, costly, and raises logistical concerns regarding animal housing. Furthermore, their advantageous small size presents a pharmacokinetic drawback, rapid renal clearance leading to a short systemic half‐life. This limits their efficacy in therapies requiring prolonged circulation and raises potential safety concerns regarding renal accumulation if conjugated to toxic payloads. Additional hurdles include their general inability to cross the blood‐brain barrier for central nervous system targeting and the use of biohazardous bacteriophage systems in their production, which complicates manufacturing and disposal. These collective limitations in production, pharmacokinetics, and safety profile necessitate ongoing research to develop strategies for mitigation and to fully realize the therapeutic potential of nanobodies across diverse diseases [118].

In conclusion, incorporating these diverse antibody fragments into nanocarriers facilitates not only improved targeting and penetration, but also modular and programmable surface functionalization, paving the way for smarter and more effective therapeutic nanoparticles.

### 3.3 Novel Antibody‐Nanomedicine Fusion Formats

In the early stage of antibody‐empowered nanomedicine development, research primarily focused on modifying the surface of nanoparticles with antibodies to confer targeting specificity. Now, numerous innovative formats have emerged, whose core idea is to integrate the targeting function of antibodies with therapeutic modules in more sophisticated and efficient modalities, giving rise to two highly representative advanced forms, antibody‐drug conjugates (ADCs) and nanobody.

ADCs often termed “magic bullets” for cancer therapy, consisting of three key components, a tumor‐targeting antibody, a stable linker that releases the drug specifically inside cancer cells, and cytotoxic payloads that induces cell death by attacking DNA, microtubules, or topoisomerase 1. This modular architecture confers a unique structural advantage by spatially decoupling the targeting and killing functions, enabling the precise delivery of ultra‐potent cytotoxic agents that would be too toxic for systemic administration alone. ADCs combine the targeting ability of monoclonal antibodies with the potent cell‐killing effects of cytotoxic drugs [119]. For instance, Golfier et al. developed anetumab ravtansine, a mesothelin‐targeting ADC that delivers the microtubule inhibitor DM4 into tumor cells, demonstrating high selectivity and potent cytotoxicity in mesothelin‐expressing models [120]. Similarly, Guerra et al. constructed CDX‐0239, an anti‐Anaplastic Lymphoma Kinase (ALK) antibody conjugated to pyrrolobenzodiazepine (PBD) dimers, which enables targeted delivery of payloads to ALK‐positive pediatric and adult tumors [121]. Auvert et al. engineered a suite of HER2‐targeted ADCs featuring an exatecan payload and optimized linkers, including a high‐DAR IgG and novel Fc‐deficient formats, which demonstrated potent efficacy against HER2‐positive breast cancer [122].

Nanobodies represent another fusion format. Given their small size, high stability, and single‐domain architecture, nanobodies are particularly suitable for therapeutic contexts that require deep tissue penetration, stability in harsh physiological environments, or modular assembly into multispecific constructs. Beyond serving as targeting ligands functionalized onto nanoparticle surfaces, nanobodies itself have also demonstrated significant potential for diverse clinical applications. For instance, Biesemann et al. developed the anti‐TNF‐α/IL‐6 bispecific nanobody, demonstrating strong therapeutic potential for rheumatoid arthritis by effectively inhibiting key disease‐relevant pathways [123]. And, Zhu et al. obtained a series of anti‐CD4 nanobodies from alpacas immunized with human CD4 protein. Among them, Nb457 displays high potency and broad‐spectrum activity against HIV‐1, surpassing conventional antibody efficacy [124]. Zhang et al. developed a PD‐L1/VEGFR2‐bispecific nanobody that demonstrated dual antitumor and antimetastatic activities [125].

## 4 Biomedical Applications of Antibody‐Empowered Nanomedicine

### 4.1 Antibody‐Empowered Nanomedicine for Rapid Diagnosis

**FIGURE 5:**
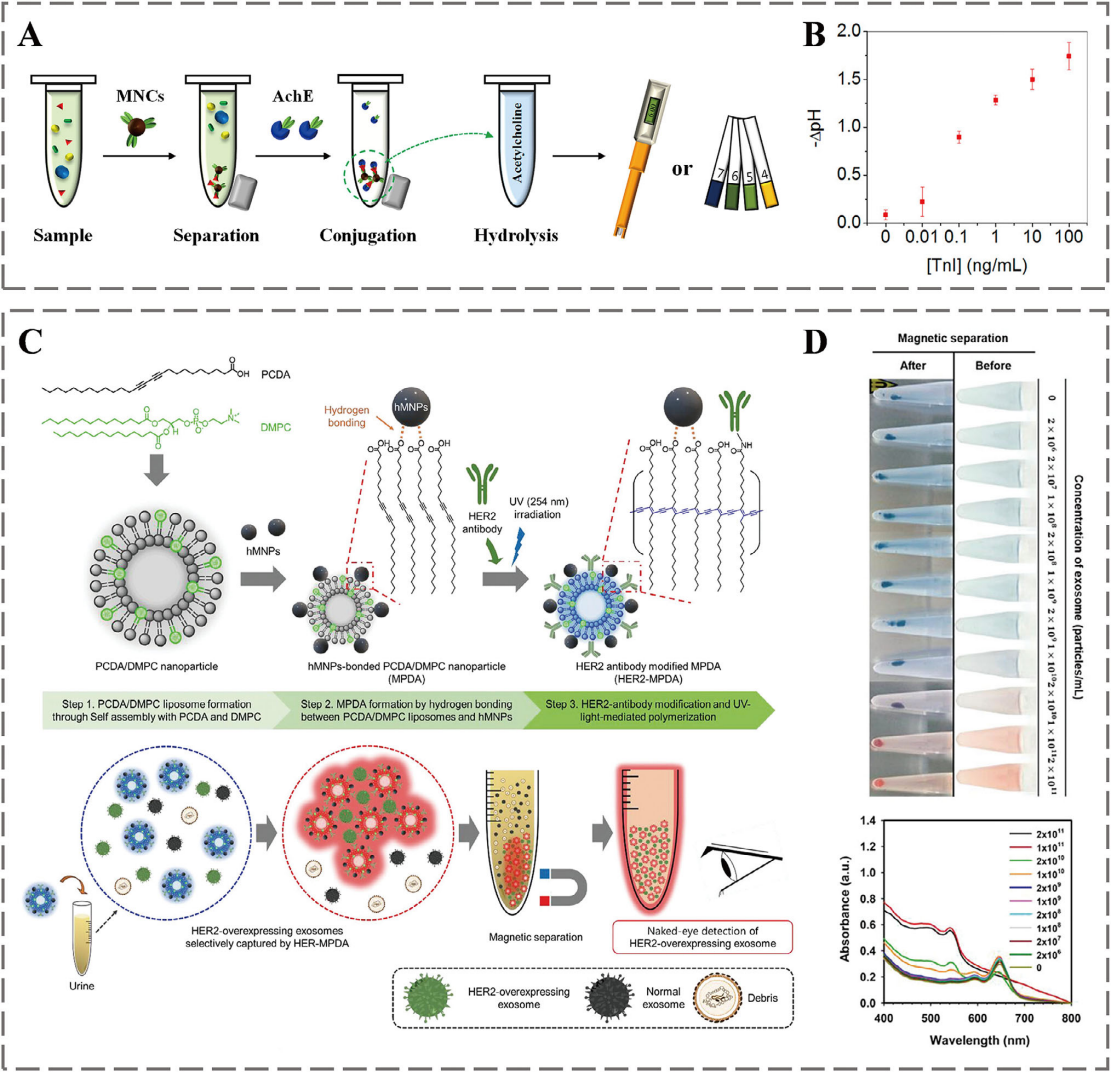
A) Diagram illustrating troponin I detection via pH measurement (meter or strips). B) Plot of pH value against troponin I concentration after a 10‐min Ach hydrolysis. Reproduced with permission [129]. Copyright 2013, American Chemical Society. C) Preparation scheme of HER2‐MPDA and its use in colorimetric assay for HER2‐positive exosomes in raw urine. D) Visual color changes and recorded absorbance spectra of HER2‐MPDA incubated with different levels of HER2‐positive exosomes. Reproduced with permission [130]. Copyright 2023, John Wiley and Sons.

Detecting specific metabolites and quantifying their level variations in different biofluids has been pivotal for understanding physiological states and diagnosing diseases. Antibody‐empowered nanomedicine is able to perform metabolite detection and quantification via metabolite‐antibody interactions, ultimately achieving rapid and accurate diagnosis, offering a treatment time window of life‐threatening diseases [126]. For instance, troponin serves as a key biomarker for cardiac injury [127]. When cardiac muscle is damaged, troponin leaks into the bloodstream, causing its concentration to surge from approximately 10 pg/mL to over 1 ng/mL within hours. Consequently, tracking troponin levels can help prevent fatalities from sudden cardiac events [128]. Based on this, Kwon et al. employed monoclonal antibody‐functionalized Fe_3_O_4_ magnetic nanoparticle clusters (MNCs) to develop a facile and sensitive method for troponin detection. As shown in Figure 5A, troponin I was selectively captured from human serum by antibody‐functionalized MNCs and magnetically isolated. The subsequent conjugation with acetylcholinesterase (AchE)‐tagged antibodies enabled real‐time, sensitive detection via the enzyme‐mediated hydrolysis of acetylcholine (Ach), which was quantified using a pH meter or colorimetric strips (Figure 5B) [129]. In another study, Kim et al. developed a sensor by conjugating anti‐HER2 antibodies to magnetic polydiacetylene (PDA) nanoparticles (Figure 5C). This design enabled the specific capture of HER2‐overexpressing exosomes and produced a visible colorimetric signal (blue‐to‐red transition) upon binding (Figure 5D). Thus, the resulting HER2‐MPDA sensor could detect target exosomes in urine, providing an equipment‐free and non‐invasive tool for diagnosing HER2‐positive breast cancer [130]. Yang et al. used antibody‐conjugated magnetic nanoparticles to quantify α‐synuclein, a well‐established biomarker for Parkinson's disease (PD) and Parkinson's disease dementia (PDD). This method achieves a broad dynamic range of 0.3 fg/mL to 310 pg/mL for assaying α‐synuclein in plasma, demonstrating great potential for diagnosing PD and PDD [131]. Together, these studies exemplify how antibody‐conjugated nanomaterials can be engineered into highly sensitive and practical diagnostic platforms, leveraging the specificity of antibodies for biomarker capture and the unique physicochemical properties of nanoparticles for signal generation and readout.

**FIGURE 6:**
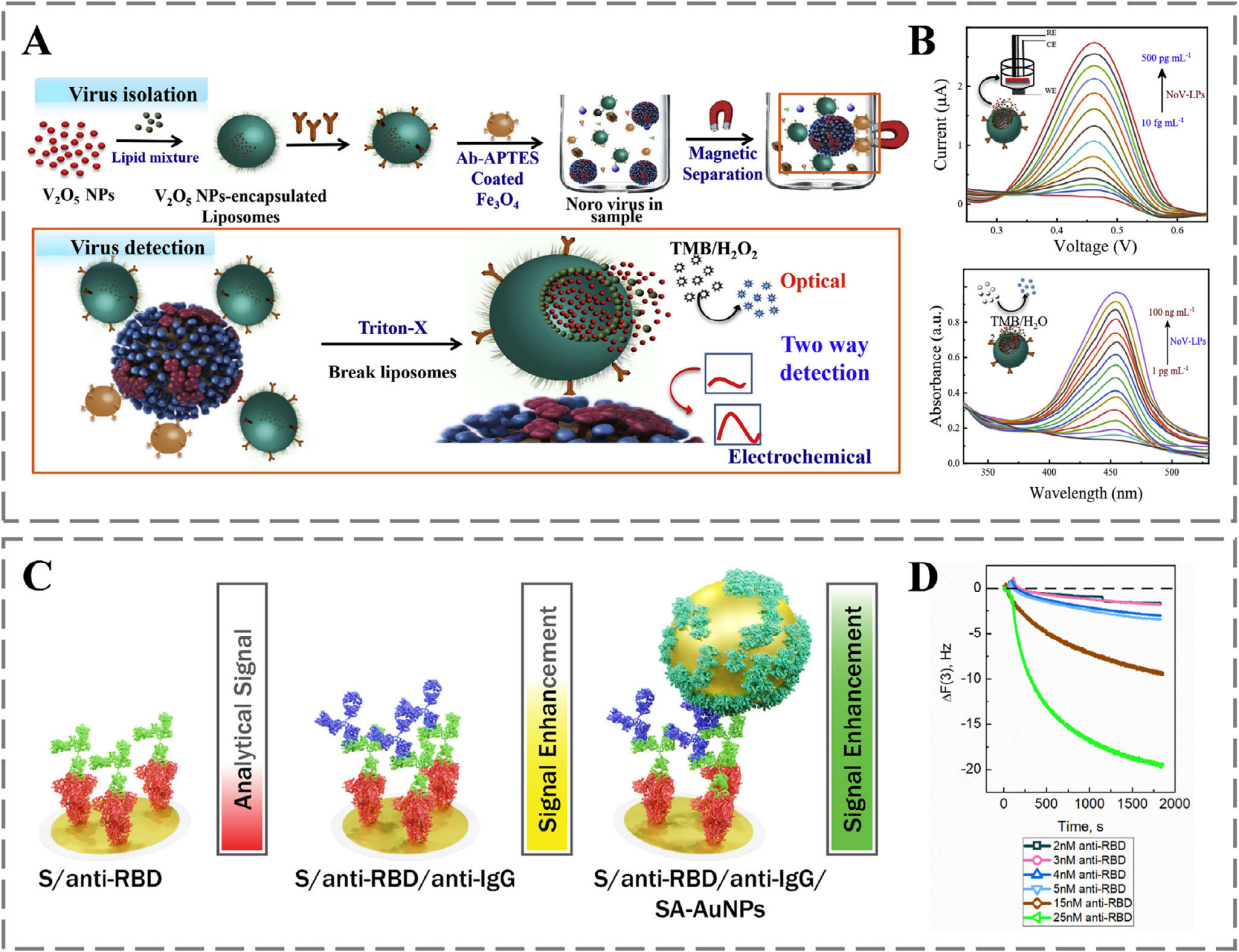
A) Schematic of the preparation procedure for VONP‐LPs. B) Dose‐response curve of the VONP‐LP dual‐modality sensor for NoV‐LP detection. Reproduced with permission [135]. Copyright 2020, Elsevier. C) Enhanced signal generation via a network of secondary antibodies and gold nanoparticles. D) Binding kinetics of anti‐RBD to immobilized SCoV2‐S, monitored by the 3rd overtone frequency shift. Reproduced with permission [136]. Copyright 2026, Elsevier.

Moreover, antibody‐empowered nanomedicine offers the potential for rapid, precise detection of various pathogens posing threats to global public health and national security [132, 133, 134]. During disease outbreaks, early pathogen identification is critical for containing transmission [133]. The high specificity of antibodies, when integrated with the signal‐enhancing properties of engineered nanomaterials, enables the development of highly sensitive and rapid diagnostic tools. This is effectively demonstrated in platforms such as the dual‐modal sensor developed by Ganganboina et al., which employs antibody‐functionalized V_2_O_5_ nanoparticle‐encapsulated liposomes (VONP‐LPs) to achieve ultrasensitive colorimetric and electrochemical detection of norovirus (Figure 6A,B) [135]. Similarly, Usvaltas et al. developed a sensitive sandwich immunoassay for SARS‐CoV‐2 antibodies using antibody‐functionalized gold nanoparticles (AuNPs) for signal amplification (Figure 6C,D) [136].

Beyond addressing immediate detection needs, a key translational advantage of these antibody‐nanomaterial platforms lies in their inherent scalability. Scalability can be realized horizontally through the modular exchange of conjugated antibodies to rapidly adapt the core sensing platform to new pathogens, and vertically through the integration of multiple detection modalities (e.g., optical, electrochemical) to suit scenarios ranging from high‐throughput labs to point‐of‐care use. A prime example of such a modular, scalable design is the work by Chen et al., who developed a rapid detection platform based on nanobody‐conjugated AuNPs [137]. The core detection mechanism, where antigen binding triggers AuNP aggregation and produces measurable optical or electrical changes, remains constant, while the system can be retargeted to a different viral antigen simply by replacing the surface‐conjugated nanobody. This design principle underscores potential of the platform as a versatile framework that can be rapidly deployed against future emerging pathogen threats.

### 4.2 Antibody‐Empowered Nanomedicine for High‐Precision Bioimaging

**FIGURE 7:**
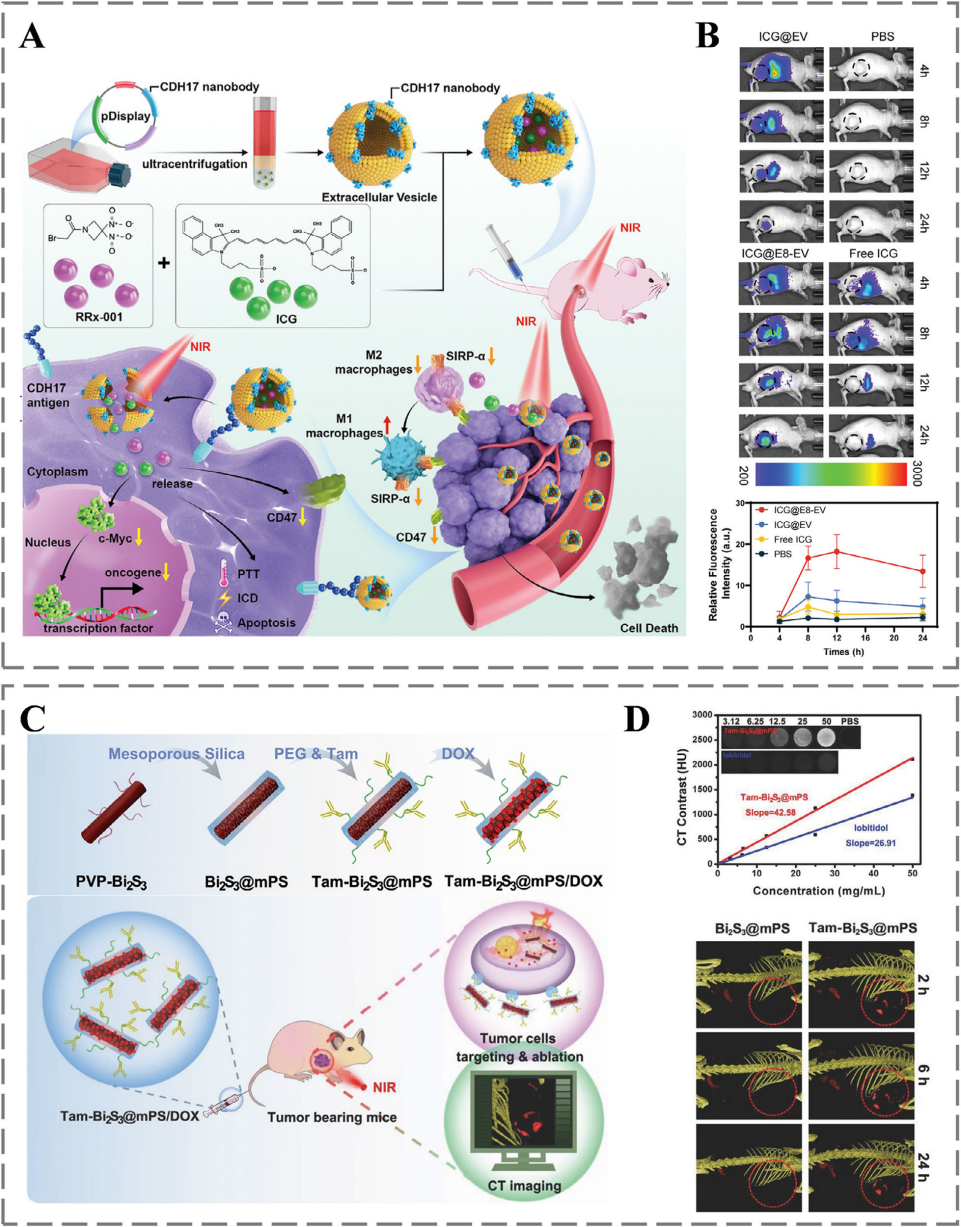
A) Design of CDH17‐targeting extracellular vesicles for theragnostic delivery and tumor‐associated macrophage polarization in gastric cancer. B) In vivo GC imaging with CDH17 nanobody‐engineered EVs. Reproduced with permission [141]. Copyright 2022, John Wiley and Sons. C) Architecture of extracellular vesicles engineered with CDH17‐specific nanobodies. D) Tam‐Bi_2_S_3_@mPS nanoparticles as a contrast agent for computed tomography across cellular and live‐animal models. Reproduced with permission [142]. Copyright 2017, John Wiley and Sons.

Antibody‐empowered nanomedicine can leverage exceptional target specificity to deliver fluorescent dyes and imaging tracers to disease sites, enabling high‐precision molecular imaging [138, 139, 140]. As illustrated in Figure 7A, Xia et al. developed cadherin 17 (CDH17)‐targeted nanobody‐engineered extracellular vesicles (E8‐EVs) for specific delivery of indocyanine green (ICG), enabling rapid imaging of gastric cancer. These vesicles were co‐loaded with ICG and the therapeutic agent RRx‐001, combining imaging and treatment capabilities. The E8 nanobody conferred high tumor‐targeting specificity, which enhanced ICG accumulation and minimized off‐target signals, as confirmed by ex vivo analysis (Figure 7B) [141]. In another example, Li et al. constructed trastuzumab‐functionalized Bi_2_S_3_@mPS/DOX nanoparticles for HER2‐positive breast cancer theranostics (Figure 7C). By conjugating trastuzumab to bismuth sulfide‐based nanoparticles, the system achieved precise tumor accumulation and enabled deep‐tissue computed tomography imaging, alongside therapeutic potential (Figure 7D) [142]. Yang et al. reported an albumin‐based nanobody system that significantly enhanced NIR‐II fluorescence imaging. The superior contrast achieved enabled precise guidance for subsequent therapeutic intervention, highlighting the critical role of high‐quality imaging in steering cancer treatment [143]. These examples collectively demonstrate that the synergy between antibody‐mediated targeting and the signal‐generating properties of nanomaterials forms the foundation for high‐contrast, disease‐specific imaging, which is indispensable for accurate diagnosis and image‐guided therapy.

### 4.3 Antibody‐Empowered Nanomedicine for Efficient Therapy of Various Diseases

As mentioned before, antibody‐empowered nanomedicine combined the intrinsic advantages of antibodies and nanomedicines, making them with multiple core benefits, including broad payload selection, high drug loading capacity, controlled drug release, precise targeting ability, and inherent immunotherapeutic potential. Therefore, they are powerful tools for highly effective disease treatment. Below, we concisely review antibody‐empowered nanomedicine applications in biomedicine for disease management, focusing on cancer, neurological disorders, inflammatory, dermatological conditions, and ocular pathologies.

#### 4.3.1 Cancer Treatment

Cancer is a major global threat to human health. Radiotherapy, chemotherapy, and surgery constitute the three primary conventional therapeutic approaches currently used in clinical practice. Despite their effectiveness, significant limitations hinder these treatments, including poor target specificity, severe adverse effects, high rates of chemoresistance, and the potential to trigger tumor metastasis [144]. Consequently, researchers are actively advancing the refinement of existing conventional therapies and the development of novel treatment strategies for cancer [145, 146, 147, 148, 149, 150].

**FIGURE 8:**
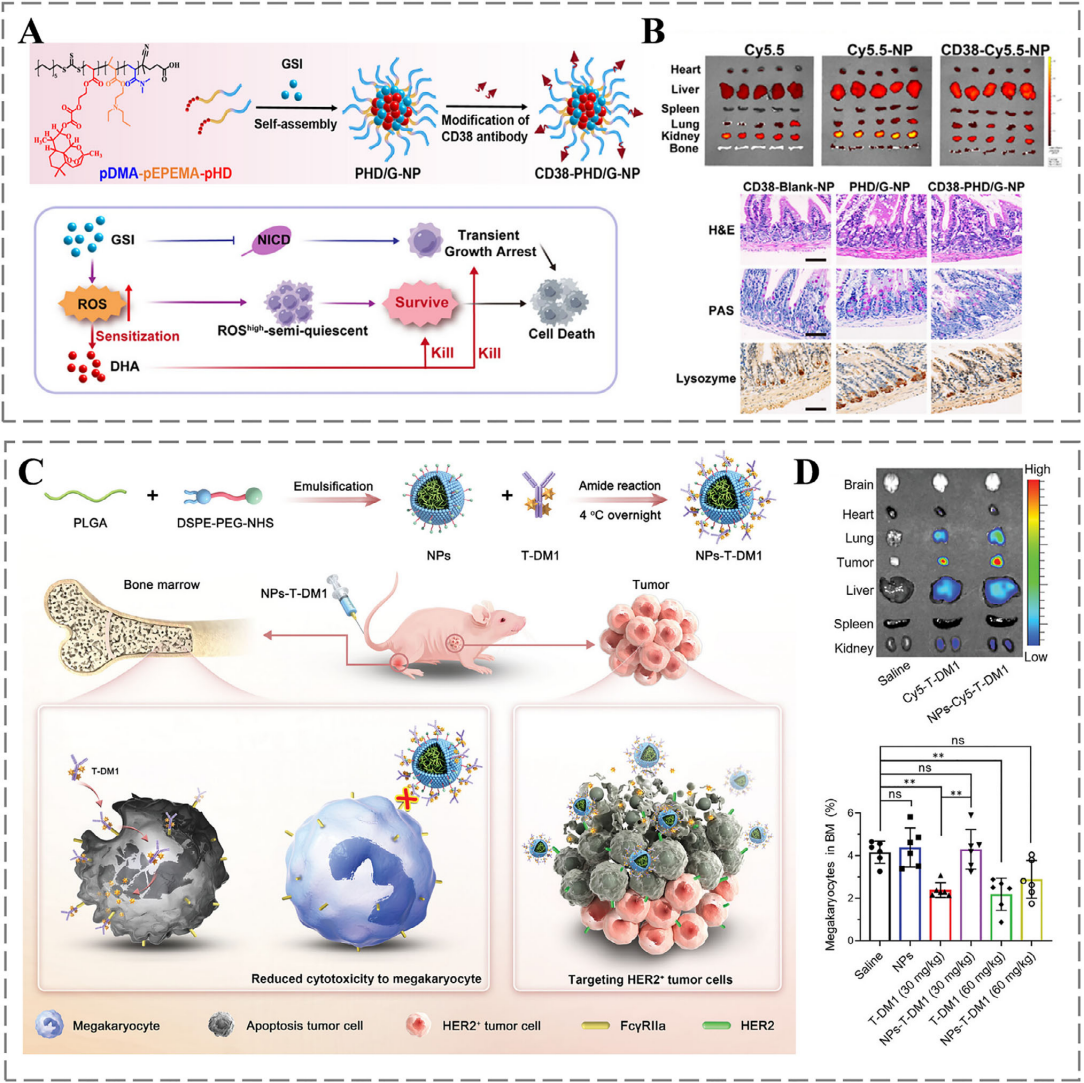
A) Design of the CD38‐PHD/G‐NP platform and the underlying DHA‐GSI synergy. B) Preferential uptake of CD38‐Cy5.5‐NPs within leukemic niches, mitigating the gastrointestinal adverse effects of GSI. Reproduced with permission [152]. Copyright 2025, John Wiley and Sons. C) Strategy for delivering T‐DM1 via nanoparticles to limit off‐target toxicity in megakaryocytes. D) NPs‐T‐DM1 exhibits enhanced tumor localization while preserving megakaryocyte viability. Reproduced with permission [153]. Copyright 2024, John Wiley and Sons.

Currently, numerous tumor‐associated antigens (TAAs) and tumor‐specific antigens (TSAs) have been identified, enabling targeted therapies against these antigens as a crucial strategy for cancer treatment [151]. In this regard, antibody‐empowered nanomedicine enables active targeted delivery through specific antibody binding to TAAs or TSAs. This antibody‐mediated targeting not only ensures precise tumor cell recognition but also minimizes off‐target damage to healthy tissues, thereby enhancing tumor‐specific cytotoxicity and improve the safety profile of cancer therapeutics. For instance, T‐cell acute lymphoblastic leukemia (T‐ALL) cells exhibit high surface expression of the CD38 antigen. Leveraging this characteristic, Jia et al. developed PHD/G‐NPs, a CD38 antibody‐modified nanotherapeutic system for targeted T‐ALL treatment (Figure 8A,B). These nanoparticles co‐deliver γ‐secretase inhibitors (GSIs) and dihydroartemisinin (DHA) to achieve synergistic chemotherapy. The CD38 antibody promotes tumor‐specific accumulation by binding CD38 on T‐ALL cells, increasing local concentrations of GSI and DHA and enhancing killing efficacy. Furthermore, targeted delivery reduces nanoparticle distribution in normal tissues, mitigating GSI‐induced damage to intestinal epithelial cells and associated gastrointestinal toxicity [152]. Similarly, Liu et al. developed a nano‐delivery system for trastuzumab emtansine (T‐DM1) by conjugating it with poly (lactic‐co‐glycolic acid) (PLGA) nanoparticles (NPs‐T‐DM1) (Figure 8C). And, NPs‐T‐DM1 demonstrated reduced binding to megakaryocytes while maintaining targeting efficacy against HER2‐overexpressing tumor cells, offering a safer and more effective strategy for treating HER2‐positive breast cancer (Figure 8D) [153].

**FIGURE 9:**
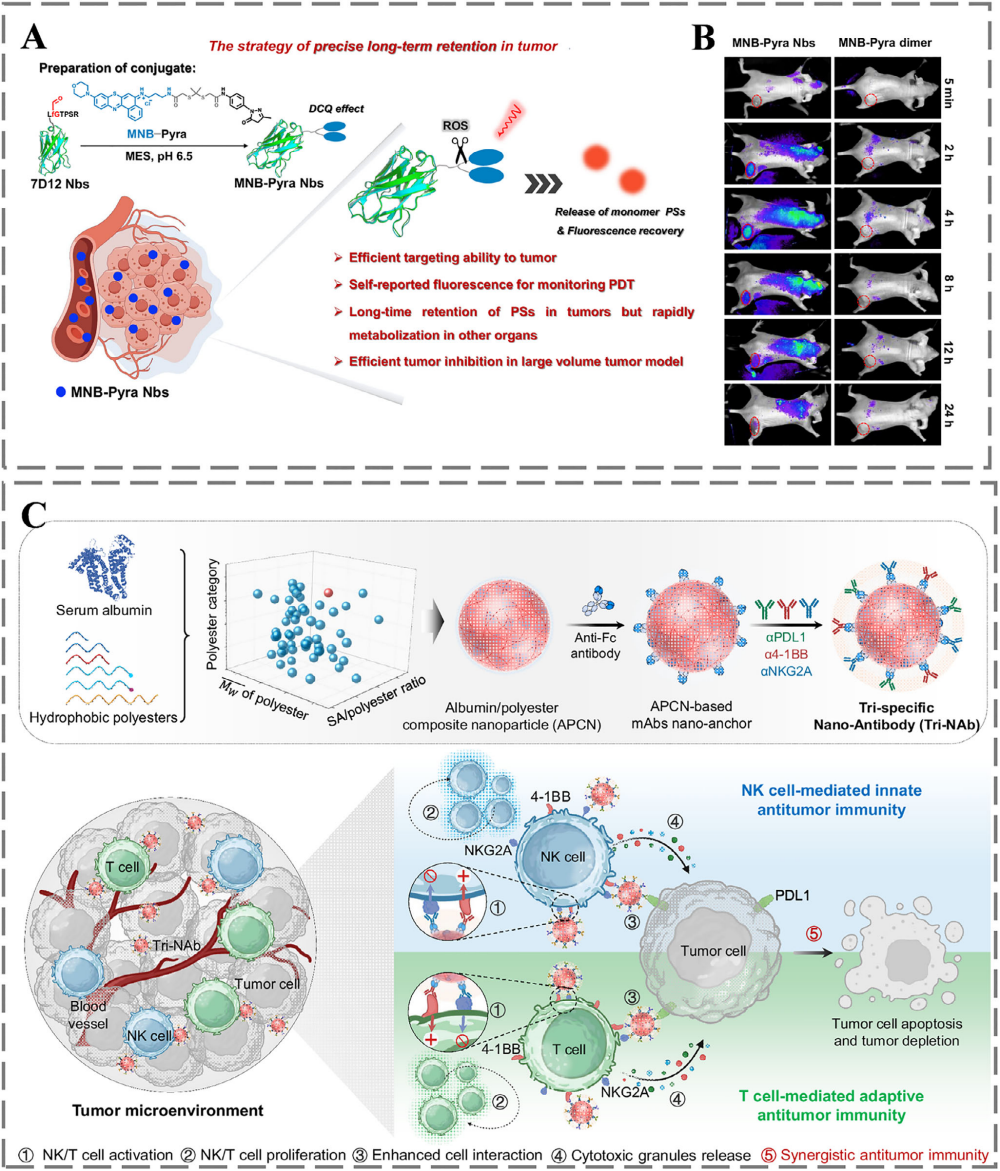
A) Assembly of a self‐reporting photodynamic nanobody conjugate enabling persistent tumor tissue retention for continuous PDT. B) Pharmacokinetics and real‐time in vivo monitoring of MNB‐Pyra dimer and MNB‐Pyra Nbs within 24 h of administration. Reproduced with permission [155]. Copyright 2024, Springer Nature. C) Architecture of the Tri‐NAb and its mechanistic role in synergizing innate and adaptive immune responses against tumors. Reproduced with permission [156]. Copyright 2024, Springer Nature.

In addition to chemotherapy, antibody‐empowered nanomedicine can also leverage novel therapeutic modalities such as photothermal therapy (PTT), photodynamic therapy (PDT), immunotherapy, and combination therapy to precisely recognize and efficiently kill tumor cells. For instance, Annušová et al. developed a novel MoOx‐based nanoconjugate functionalized with M75 antibodies targeting the tumor hypoxia marker CAIX for selective tumor PTT. Upon near‐infrared irradiation, these conjugates induced significant photothermal heating, demonstrating the potential of a hypoxia‐targeted strategy for tumor ablation [154]. Chen et al. leveraged EGFR‐targeting nanobodies (7D12) to deliver the type I photosensitizer (MNB‐Pyra) to EGFR‐overexpressing tumor cells, enabling efficient tumor targeting, sustained intratumoral retention, and effective PDT treatment (Figure 9A,B) [155]. Additionally, Ye et al. developed a tri‐specific nano‐body (Tri‐NAb) using an optimized albumin/polyester composite nanoparticle (APCN), and incorporates antibodies against PDL1, 4‐1BB, and NKG2A (Figure 9C). As an innovative immunotherapy strategy, the Tri‐NAb promotes the proliferation and activation of both NK cells and CD8^+^ T cells, enhances their interactions with tumor cells, and boosts cytotoxic granule release, thereby mediating potent tumor cell killing [156].

**FIGURE 10:**
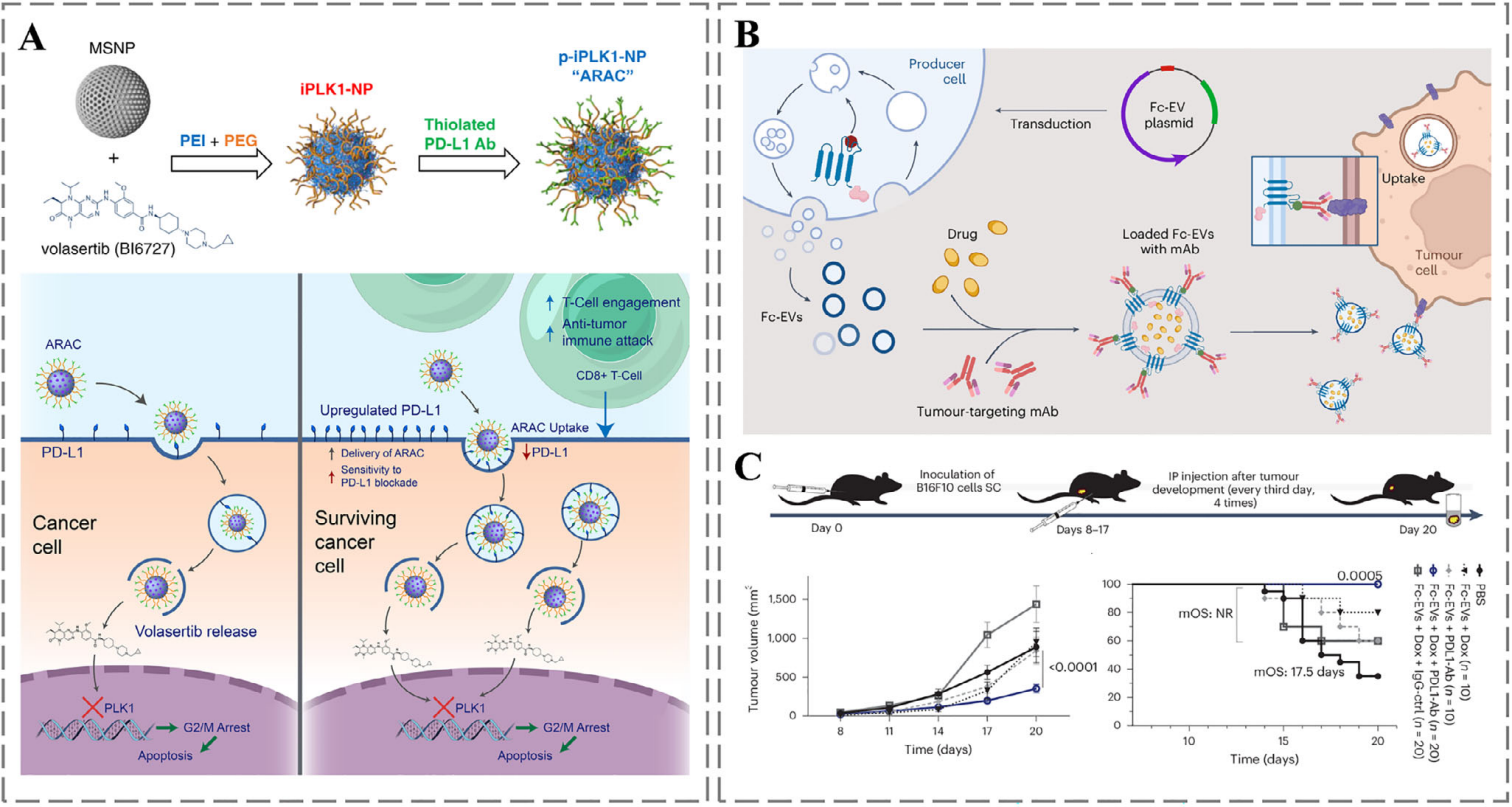
A) Fabrication scheme and working mechanism of the p‐iPLK1‐NP construct. Reproduced with permission [160]. Copyright 2022, Springer Nature. B) Engineering cells to produce extracellular vesicles functionalized with an Fc‐binding domain. C) In vivo therapy schedule for B16F10 melanoma‐bearing mice, with treatments administered every 72 h over 20 days. Reproduced with permission [161]. Copyright 2024, Springer Nature.

Recent research efforts increasingly focus on combination therapy, which refers to combination of chemotherapy, PDT, PTT, and immunotherapy. By leveraging synergistic effects, this approach significantly enhances antitumor efficacy while mitigating the adverse effects associated with monotherapy, ultimately aiming to improve long‐term patient outcomes such as survival rates [157, 158, 159]. For instance, Reda et al. developed a nanoparticle system (ARAC) co‐delivering a PLK1 inhibitor and PD‐L1 antibodies for synergistic immunotherapy (Figure 10A). Critically, the surface‐conjugated PD‐L1 antibody serves a dual purpose it enables tumor‐specific targeting and, upon internalization, blocks the therapy‐induced upregulation of PD‐L1 to prevent immune escape and activate T‐cells. This coordinated strategy of targeted delivery and immune checkpoint blockade is designed not only to shrink tumors but also to establish a durable anti‐tumor immune response, which is a critical factor for prolonging survival and preventing relapse [160]. Similarly, Wiklander et al. engineered antibody‐displaying extracellular vesicles (Fc‐EVs) for combination therapy (Figure 10B,C). This platform exemplifies the immunotherapy and chemotherapy synergy, the surface‐displayed anti‐PD‐L1 antibody blocks the PD‐1/PD‐L1 immunosuppressive pathway to activate antitumor immunity, which works cooperatively with the chemotherapeutic drugs loaded within the EVs for enhanced tumor elimination. Such a multi‐pronged attack, simultaneously targeting cancer cells and the immunosuppressive microenvironment, represents a promising strategy to overcome treatment resistance and achieve more sustained therapeutic efficacy, thereby improving progression‐free survival [161].

Beyond intrinsic synergy engineered within a single nanoplatform, a highly impactful strategy involves the extrinsic combination of antibody‐empowered nanomedicine with conventional therapeutic agents. This approach leverages the distinct and complementary mechanisms of action of each component to achieve superior outcomes compared to either agent alone, a principle with direct implications for improving clinical survival metrics. For instance, the phase Ib DS8201‐A‐U105 trial, where the combination of the ADC trastuzumab deruxtecan (T‐DXd) and the PD‐1 inhibitor nivolumab yielded a confirmed objective response rate of 65.6% in patients with HER2‐positive metastatic breast cancer. The synergy arose from T‐DXd inducing immunogenic cell death and recruiting immune cells, while nivolumab concurrently blocked the PD‐1 pathway to reinvigorate T‐cell immunity [162]. Beyond such promising individual trials, a recent systematic review encompassing 16 clinical trials across various solid tumors provides robust, aggregated evidence for this combinatorial strategy. The analysis reported a pooled objective response rate of 48.8% and a complete response rate of 9.0% for ADC‐immune checkpoint inhibitors (ICIs) combinations, quantitatively confirming their superior efficacy over many monotherapy strategies. Importantly, while the overall safety profile of the combinations was found to be manageable and comparable to ADC monotherapy, the analysis highlighted the need for vigilant monitoring of specific overlapping toxicities, such as interstitial lung disease, which underscores the importance of careful clinical management when implementing these potent extrinsic combinations [163].

#### 4.3.2 Neurological Disorders Treatment

**FIGURE 11:**
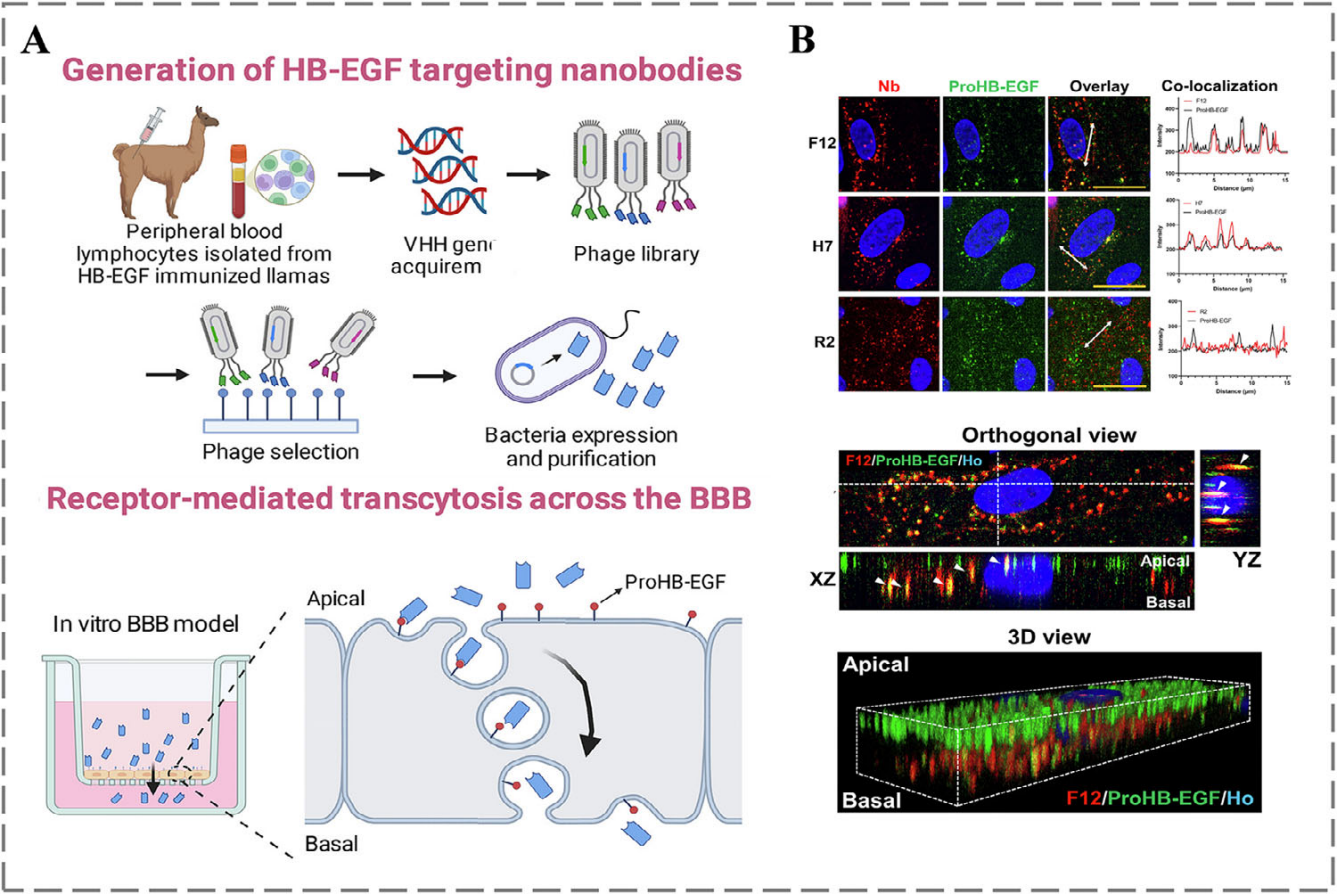
A) Screening and in vitro characterization of high‐affinity anti‐proHB‐EGF nanobodies (F12, H7) for their potential to cross the blood‐brain barrier. B) 3D spatial analysis confirming the co‐localization and traversal of proHB‐EGF‐Nb complexes during transcytosis across a synthetic blood‐brain barrier. Reproduced with permission [172]. Copyright 2025, Elsevier.

Neurological disorders are a major and increasing global health challenge, which accounts for a substantial portion of the disease burden worldwide. Among them, neurodegenerative disorders, notably Alzheimer's disease (AD), Parkinson's disease (PD), and cerebrovascular events, particularly stroke, have attracted considerable attention, due to their steadily increasing incidence, and the persistent limitations in treatment efficacy [164, 165, 166, 167]. However, the development of new therapies has been hindered by the existence of the blood‐brain barrier (BBB), which composed of tightly joined, non‐fenestrated endothelial cells and regulated by specialized junctions, support cells, and efflux transporters, maintains brain homeostasis by selectively controlling molecular influx and efflux [168, 169]. Fortunately, the corporation of targeting ligands, such as antibodies, facilitates traversal across the blood‐brain barrier (BBB) [170, 171]. As shown in Figure 11A, Qiu et al. focused on BBB drug delivery and identified two nanobodies (F12 and H7) targeting the transmembrane precursor of heparin‐binding EGF‐like growth factor (proHB‐EGF), which is expressed on the intraluminal side of brain endothelial cells. These nanobodies cross the BBB via receptor‐mediated transcytosis (RMT) and exhibit favorable safety profiles, thereby offering novel strategies for targeted drug delivery in Neurological disorders (Figure 11B) [172]. Recently, Oosterlaken et al. developed DN13‐DN1, a bivalent biparatopic nanobody composed of two camelid heavy‐chain antibodies, which can cross the BBB to bind to and potentiate the homodimeric metabotropic glutamate receptor 2 (mGlu2) after peripheral administration. DN13‐DN1 demonstrates therapeutic potential by rescuing cognitive and sensorimotor gating deficits caused by NMDA receptor hypofunction, validating nanobodies as a promising approach for targeting brain receptors and treating brain disorders [173].

#### 4.3.3 Inflammatory Diseases Treatment

Inflammation is a vital defense mechanism against harmful stimuli like pathogens, tissue damage, toxins, and physical trauma [174, 175]. Dysregulation of this process is a key driver of numerous acute and chronic diseases, including acute organ injuries (liver, lung), asthma, inflammatory bowel disease (IBD), rheumatoid arthritis (RA), atherosclerosis, and neurodegenerative disorders [176, 177, 178, 179]. Anti‐inflammatory therapy has thus emerged as a promising strategy for treating the aforementioned diseases. However, clinical implementation of anti‐inflammatory therapy faces substantial challenges including poor drug stability, inadequate targeting capability, short retention time at lesion sites, and significant side effects, all of which compromise therapeutic efficacy [180, 181, 182, 183]. To overcome these limitations, antibody‐empowered nanomedicine combines the specificity of antigen‐antibody interactions with the intrinsic advantages of nanocarriers (e.g., stimulus‐responsive drug release and enhanced biocompatibility), thereby improving delivery precision. Such antibody‐functionalized systems represent a versatile platform for the targeted treatment of inflammatory diseases.

**FIGURE 12:**
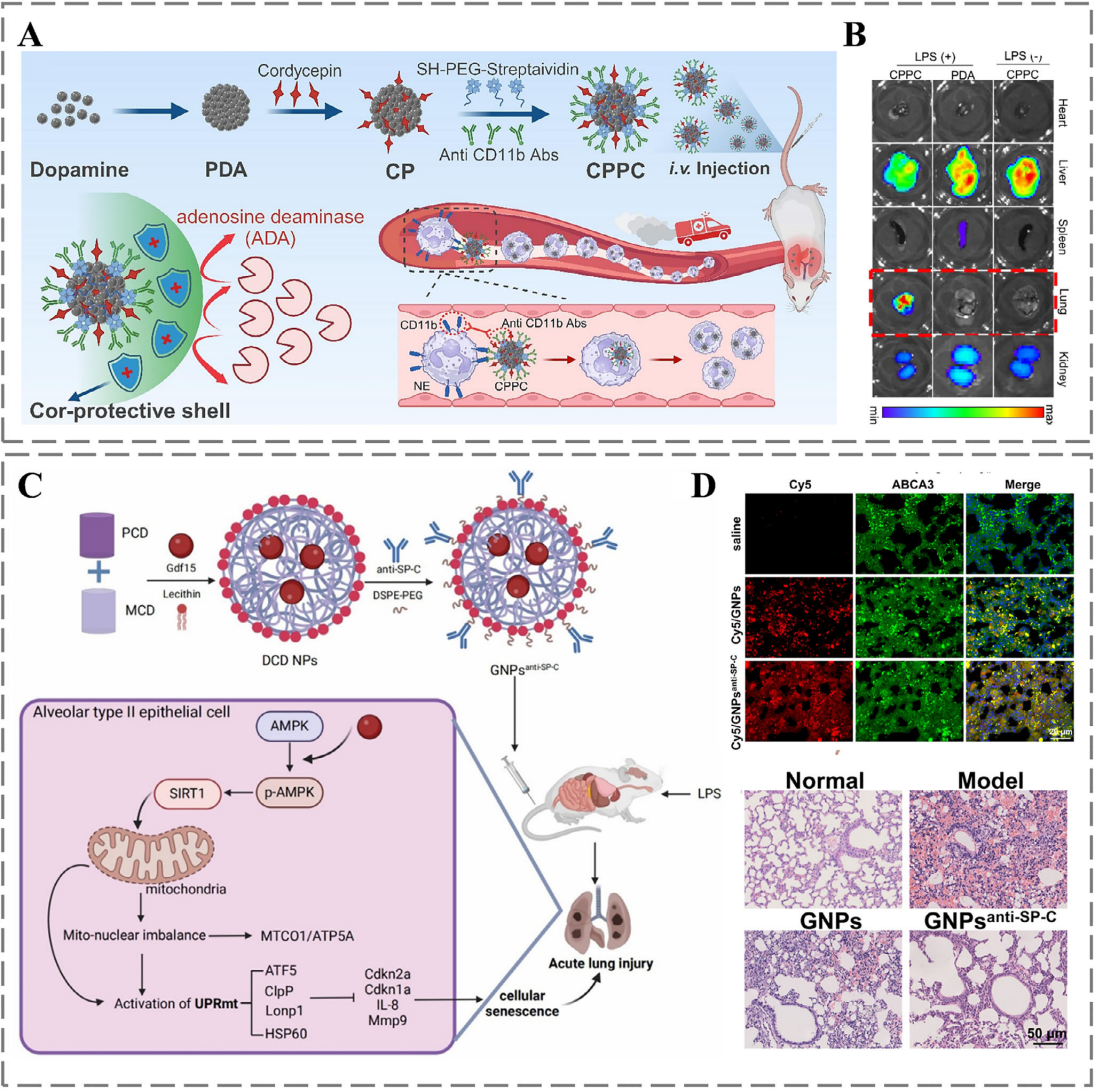
A) Design and synthesis of the CPPC platform for conferring protective effects in lung‐targeted treatment. B) Ex vivo biodistribution analysis: fluorescence signals in harvested organs following IVIS. Reproduced with permission [184]. Copyright 2025, Elsevier. C) Engineered GNPsanti‐SP‐C nanoparticles mitigate ACE‐II cell senescence via targeted GDF15 delivery. D) Co‐localization of NPs with ACE‐II cells (ABCA3 marker) and corresponding histopathological assessment (H&E) of lung tissues from all experimental groups. Reproduced with permission [185]. Copyright 2025, Springer Nature.

As shown in Figure 12A,B, Gao et al. recently developed neutrophil‐targeted nanoparticles (CPPC), which encapsulates cordycepin (Cor) within polydopamine (PDA) nanoparticles and is surface‐modified with anti‐CD11b antibodies, for treating acute lung injury (ALI). During acute lung injury, complex inflammation triggers the release of inflammatory mediators, and recruits circulating neutrophils to perform key immune defense and regulatory roles. The CPPC nanoparticle platform, modified with anti‐CD11b antibodies, specifically binds CD11b molecules highly expressed on neutrophils. This enables efficient delivery of CPPC nanoparticles to inflamed sites alongside the recruited neutrophils, demonstrating significant therapeutic efficacy and offering a promising new strategy for treating ALI [184]. Moreover, alveolar type II epithelial cells (ACE‐II cells) play a pivotal role in ALI progression. Their specific expression of surfactant protein C (SP‐C) not only serves as a defining marker but also makes them an ideal therapeutic target. L. Gao et al. engineered pH/ROS dual‐responsive nanoparticles (GNPsanti‐SP‐C) modified with anti‐SP‐C antibody to deliver Growth Differentiation Factor 15 (GDF15) specifically to ACE‐II cells (Figure 12C,D). In LPS‐induced ALI mice, these nanoparticles significantly reduced lung injury and inflammation while improving function [185].

Beyond acute lung injury, antibody‐functionalized nanomedicine has shown promising applications in other inflammatory diseases through distinct targeting mechanisms. In inflammatory bowel disease (IBD), for instance, an orally delivered, protease‐resistant single‐domain antibody (VHH) targeting IL‐23R (the receptor for the cytokine IL‐23) has been developed. By leveraging the unique properties of this engineered VHH, the formulation maintains stability throughout the gastrointestinal tract and effectively neutralizes a key inflammatory pathway locally, demonstrating the potential of antibody‐mediated localized interception of disease drivers [186]. Based on the central role of IL‐23, inhibitors of this pathway have emerged as a cornerstone in the clinical management of IBD [187]. A prime example is SOR102, an oral bispecific nanobody targeting both TNF and IL‐23p19. Initial clinical trials reported a favourable safety profile with minimal systemic exposure and preliminary efficacy, supporting its further development for ulcerative colitis and exemplifying the translational progress of orally active antibody fragments [188].

Similarly, in rheumatoid arthritis (RA), where TNF‐α and IL‐6 represent cornerstone therapeutic targets, antibody‐enabled nanomedicine is progressing from innovative delivery systems to clinical validation. One advanced preclinical study involves integrating a bispecific fenobody targeting both TNF‐α and IL‐6R into a GelMA microneedle system for sustained transdermal delivery. This platform exemplifies how dual‐pathway inhibition and controlled drug release can be synergistically combined to suppress inflammation and promote joint repair [189]. The translational potential of such nanobody‐based approaches is strongly supported by clinical reality. Notably, ozoralizumab (a trivalent anti‐TNFα nanobody) has achieved regulatory approval for RA treatment in several countries, concretely validating the clinical feasibility and efficacy of engineered nanobody formats in this disease [190].

Additionally, Zhu et al. developed the inhaled bivalent nanobody LQ036, targeting IL‐4Rα with high affinity, demonstrates superior efficacy in blocking IL‐4/IL‐13 signaling and attenuates allergic inflammation in a humanized mouse model, supporting its ongoing clinical development as a novel inhaled biologic for asthma [191].

#### 4.3.4 Dermatological Conditions Treatment

The skin serves as a vital protective barrier for the body, defending against harmful external substances. Data shows that the prevalence rate of skin diseases in China ranges from 40% to 70%, and dermatological conditions have become one of the common diseases endangering health and quality of life. Psoriasis is a chronic immune‐mediated disease characterized by erythematous and scalous skin lesions, with a global prevalence of 2%–3% [192, 193, 194]. The neutrophil‐targeting approach represents a promising option for psoriasis therapy. Lin et al. cleaved the anti‐mouse neutrophil antibody NIMP‐R14 into monovalent fragments to construct monovalent antibodies, and conjugated it on the surface of nanoparticles to lead precise uptake by human and mouse neutrophils. Meanwhile, nanoparticles were encapsulated with roflumilast (RFL), a phosphodiesterase (PDE) 4 inhibitor, achieving restrain inflammation and neutrophil migration. Finally, achieve precise and efficient treatment of psoriasis [195]. Beyond leveraging antibody fragments for targeted delivery, the full potential of antibody‐empowered nanomedicine is further exemplified by antibody‐drug conjugates (ADCs) in treating other refractory skin diseases. For instance, a phase II trial of the anti‐CD30 ADC brentuximab vedotin in patients with refractory diffuse cutaneous systemic sclerosis (dcSSc, *n* = 11) yielded significant improvements in skin thickness (measured by mRSS) and lung function. This success highlights the transformative promise of ADC therapy even for severe, fibrotic skin conditions like scleroderma [196].

#### 4.3.5 Ocular Disorders Treatment

In addition to the disease types discussed above, antibody‐empowered nanomedicine holds significant potential for therapeutic studies across multiple other diseases, including ocular disorders. Notably, corneal neovascularization (CorNV) is the second leading cause of blindness worldwide and severely impacts patients’ quality of life [197, 198, 199]. Currently, the most effective treatment strategy for CorNV involves targeting vascular endothelial growth factor (VEGF) [200, 201, 202]. However, effective drug delivery to the eye remains a major challenge due to several protective ocular barriers. The tightly packed corneal epithelium, for instance, limits nanoparticle penetration, while rapid precorneal clearance results in the loss of about 95% of topically administered drugs, substantially reducing therapeutic bioavailability [203]. Conventional eye drops suffer from short residence time, whereas invasive intravitreal injections carry risks of infection and retinal detachment [204]. Moreover, achieving sufficient targeting specificity toward pathological neovessels remains an ongoing hurdle in precise CorNV therapy.

**FIGURE 13:**
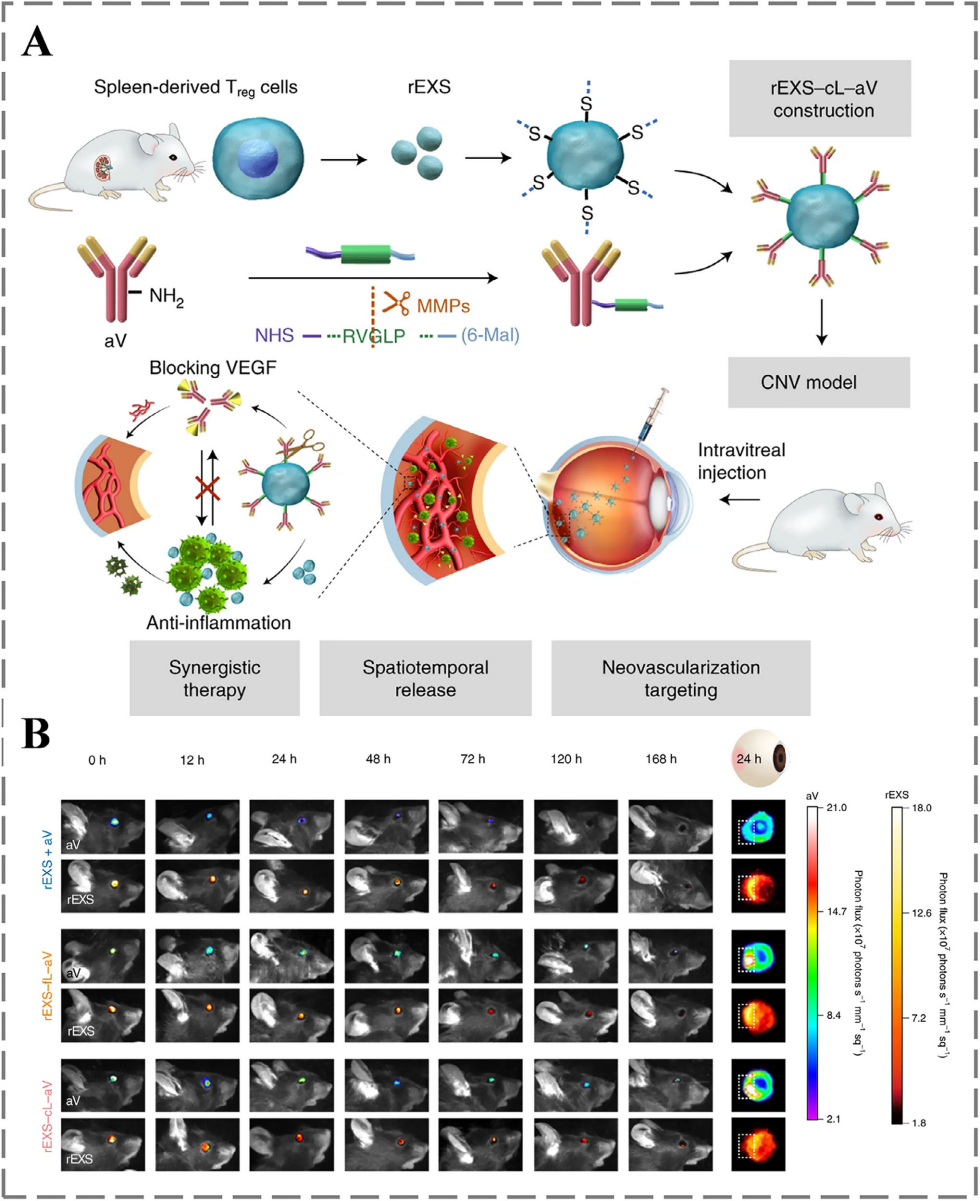
A) Fabrication scheme of rEXS‐cL‐aV and the subsequent study design for evaluating combination therapy in CNV mice. B) Time‐course in vivo imaging showing fluorescence signals in the CNV model following intravitreal administration of different rEXS formulations. Reproduced with permission [206]. Copyright 2021, Springer Nature.

To address these challenges, recent studies have focused on designing advanced nanocarriers with improved corneal permeability, prolonged ocular retention, and active targeting capabilities. For example, Liu et al. developed neutrophil‐derived nanovesicle (NCCR) eye drops, which leverage the natural inflammation‐targeting properties of neutrophil membranes to enhance lesion accumulation. Surface modification with ranibizumab enables NCCR to specifically bind and inhibit VEGF, thereby suppressing vascular endothelial cell activation and proliferation. Furthermore, NCCR combines anti‐inflammatory effects with chemiexcited photodynamic therapy (PDT), offering a multifaceted strategy that also aims to overcome ocular bioavailability limitations [205]. In another approach, Tian et al. constructed a dual‐targeting nanodrug (rEXS‐cL‐aV) by conjugating regulatory T cell‐derived exosomes (rEXS) with anti‐VEGF antibodies (aV) via a matrix metalloproteinase (MMP)‐cleavable linker (cL) (Figure 13). The rEXS platform inherently homes to inflammatory sites, thereby addressing both VEGF signaling and the inflammatory microenvironment, while the cleavable linker enables responsive drug release at the disease site. This design represents a promising strategy to improve targeting precision and therapeutic efficacy in corneal neovascular diseases [206].

Future research in ocular nanomedicine may further explore biomimetic carriers derived from endogenous cells, smart release systems responsive to pathological stimuli, and combination strategies that simultaneously enhance penetration, retention, and targeting to overcome the persistent barriers in topical corneal drug delivery.

## 5 Challenges and Outlook

Antibody‐empowered nanomedicine represents an innovative and promising strategy in the development of precision medicine. To date, a growing number of antibody‐empowered nanomedicines have advanced into clinical trials, as evidenced by the over 200 ADCs currently in clinical trials across phases I–III [207]. A notable and recent example is SOB‐100, an investigational drug consisting of exosomes functionalized with anti‐HLA‐G nanobodies for targeted delivery. This candidate is scheduled to enter a Phase I dose‐escalation clinical trial (NCT07219940) in October 2025, to assess its tolerability, safety, and pharmacokinetics in healthy subjects, marking its rapid transition from preclinical development to clinical evaluation [208]. Another compelling example is the nanobody sonelokimab, which dually inhibits IL‐17A and IL‐17F for psoriatic arthritis and has demonstrated positive efficacy and safety in a Phase II trial. These promising results have directly supported its advancement into two global Phase III clinical studies [209]. Furthermore, the ADC patritumab deruxtecan (HER3‐DXd) exemplifies successful clinical translation, having demonstrated a promising overall response rate with manageable tolerability in a Phase II trial for pretreated metastatic breast cancer, with findings now paving the way for further studies [210].

**FIGURE 14:**
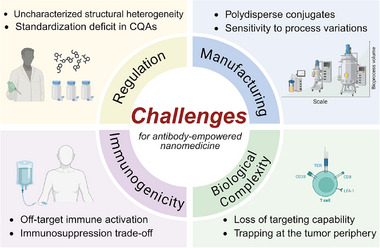
Schematic illustration of challenges of antibody‐empowered nanomedicine. Figure created with BioRender.com.

Despite numerous antibody‐empowered nanomedicines entering clinical evaluation, only a handful proceed through late‐phase trials and are approved for the market. A prime example of this translational challenge is found in ADCs. Although the clinical ADC pipeline has expanded remarkably to encompass over 200 investigational agents, fewer than 20 have obtained regulatory approval for market entry. This stark contrast between the robust pipeline and the limited number of marketed therapies underscores the significant attrition rate in the field [211]. Similarly, this pattern is mirrored in the field of nanobodies. Although a considerable and growing number of nanobody‐based candidates have advanced into clinical trials, only four have achieved global marketing approval to date. This further exemplifies the steep translational hurdle in bringing antibody‐empowered nanomedicines to patients [212, 213]. Here, we systematically explore the fundamental challenges and critical limitations that must be addressed to advance preclinical studies and facilitate their translation into clinical practice (Figure 14) [214].

First, antibody‐empowered nanomedicine faces a distinct and formidable regulatory hurdle that stems from a fundamental lack of structural definition. The therapeutic efficacy and safety of these complex conjugates are dictated not just by the identity of their components but by their precise architecture, the spatial arrangement, orientation, and integrity of the antibody on the nanoparticle surface. However, they are often characterized by average physicochemical values, leaving critical structural and functional variations between individual particles undefined. This ambiguity directly conflicts with the rigorous quality control principles demanded for regulatory approval. Specifically for antibody conjugates, while studies routinely confirm successful functionalization, key critical quality attributes, such as controlled ligand orientation, preserved antibody integrity, and consistent immunoreactivity, are frequently overlooked and lack standardized assessment. This gap generates unacceptable batch‐to‐batch variability, undermining the robust reproducibility required by agencies like the FDA and creating a significant barrier to the translational pathway of these promising platforms [4, 215].

Second, the translation of antibody‐empowered nanomedicine is critically hampered by formidable manufacturing challenges, where the demand for precise structural control collides with the realities of scalable production. Unlike conventional agents, the synthesis of these conjugates requires mastering a dual‐aspect engineering feat, the reproducible fabrication of nanoparticle cores with optimal and consistent physicochemical properties (size, morphology, surface charge), and the precise, oriented conjugation of antibodies that preserves their integrity and functionality. Current synthesis methods often struggle with this complexity, yielding heterogeneous products with high polydispersity. While advanced technologies like microfluidics and template‐based synthesis offer promising pathways toward monodisperse nanoparticles and more reproducible bioconjugation, standardizing these intricate, multi‐step processes under Good Manufacturing Practice (GMP) remains a significant hurdle. The inherent sensitivity of both nanoparticle synthesis and antibody coupling strategies to minor variations makes achieving rigorous batch‐to‐batch consistency a central obstacle to the widespread clinical adoption of these advanced therapeutics [214].

Third, the clinical translation of antibody‐empowered nanomedicine is critically challenged by the complex and multifaceted issue of immunogenicity. This risk arises not only from the conjugated antibody itself but is significantly amplified by the nanocarrier platform, which can enhance the antigenicity of surface ligands and provoke unintended immune recognition against the entire construct. Such immune responses may lead to the generation of anti‐drug antibodies that can neutralize therapeutic function, alter pharmacokinetics, and potentially cause adverse events. Conversely, strategies employed to suppress this immunogenicity must be carefully calibrated, as they might inadvertently impair essential interactions with the immune system. Therefore, advancing these platforms into clinical application necessitates a dual focus, the precise engineering of conjugates to minimize immune recognition, coupled with comprehensive immunotoxicity profiling throughout development. This balanced approach is essential to ensure that the therapeutic efficacy of antibody‐nanoparticle conjugates is not undermined by safety concerns, thereby fulfilling the stringent requirements for regulatory approval and successful clinical use [216, 217].

Finally, despite sophisticated targeting designs, only a minimal fraction of antibody‐empowered nanomedicine successfully accumulates at the intended disease site. This inefficiency stems from a series of sequential biological barriers that systematically compromise targeting efficacy. Upon intravenous administration, nanoparticles rapidly form a protein corona that can obscure conjugated antibodies and nullify their active targeting capability. Even upon reaching the tumor vasculature, a high‐affinity antibody can itself become a barrier through the “binding‐site barrier” effect, causing premature trapping of nanoconjugates on perivascular cells and severely restricting deep tissue penetration. Therefore, to overcome these barriers, the nanoconjugate must be engineered as an integrated system, optimized for navigation through complex physiological environments rather than for isolated properties in vitro. This systemic approach is critical for translating promising efficacy into clinical success [40].

## 6 Conclusions

In conclusion, this review has comprehensively examined the antibody functionalization strategies for nanomedicine, classification of antibody‐empowered nanomedicine, and the significant current progress in their development. We have highlighted their promising biological applications across rapid diagnostics, precise bioimaging, and efficient therapy for various diseases, including cancer, neurological disorders, inflammatory diseases, dermatological conditions, and ocular pathologies. While substantial progress has been made, the development of antibody‐empowered nanomedicine remains in its preliminary stage, and its clinical translation faces critical challenges. Encouragingly, driven by advances in biotechnology, antibody‐empowered nanomedicine is poised to shorten the critical path from preclinical discovery to clinical implementation, paving the way for more personalized, effective, and minimally invasive approaches to managing a wide array of human diseases.

## Author Contributions

J.Z., Q.W., F.L., and C.C. gave the conceptualization. C.C. conducted the investigation and wrote – original draft. X.C., Z.G., M.S., and Y.Y. created the specific visualization. All the authors revised the manuscript. J.Z. and Q.W. contributed to Funding acquisition.

## Conflicts of Interest

The authors declare no conflicts of interest.

## Data Availability

The authors have nothing to report.
